# Optical polarization analogs in inelastic free-electron scattering

**DOI:** 10.1126/sciadv.adj6038

**Published:** 2023-12-20

**Authors:** Marc R. Bourgeois, Austin G. Nixon, Matthieu Chalifour, David J. Masiello

**Affiliations:** ^1^ Department of Chemistry, University of Washington, Seattle, WA 98195, USA.; ^2^ Department of Physics, University of Washington, Seattle, WA 98195, USA.

## Abstract

Advances in the ability to manipulate free-electron phase profiles within the electron microscope have spurred development of quantum-mechanical descriptions of electron energy loss (EEL) processes involving transitions between phase-shaped transverse states. Here, we elucidate an underlying connection between two ostensibly distinct optical polarization analogs identified in EEL experiments as manifestations of the same conserved scattering flux. Our work introduces a procedure for probing general tensorial target characteristics including global mode symmetries and local polarization.

## Teaser

A theoretical description of optical polarization analogs in phase-shaped electron energy loss measurements is introduced.

## INTRODUCTION

Inherently necessitating the exchange of both energy and momentum, measurements involving the absorption and scattering of optical waves and energetic particles provide a wealth of information characterizing atomic, molecular, and nanoscale systems. Selection rules based on the optical polarization degrees of freedom, in particular, are indispensable tools for probing target excitation symmetries, albeit with spatial resolution limited by optical diffraction. With their atomic-scale de Broglie wavelength, high-energy (100 to 200 keV) electrons in the scanning transmission electron microscope (STEM) offer superior spatial localization but lack polarization degrees of freedom because they are accurately described by the scalar Schrödinger equation. Despite this deficiency, it was shown early on that transverse linear momentum (LM) transfer ℏ**q**_⊥_ in widefield near-edge electron energy loss (EEL) processes could be exploited to selectively probe atomic inner shell excitations with distinct symmetries (*1*) and more recently to probe transversely polarized electric fields on the atomic length scale (*2*). A formal connection between the photon polarization $\hat{\epsilon }$ in x-ray absorption measurements and ℏ**q**_⊥_ during core-loss inelastic electron scattering measurements was established in the electrostatic limit (*3*–*7*), culminating in the experimental realization of magnetic circular dichroism measurements within a TEM (*8*, *9*).

Improved monochromation and aberration correction technologies, on the other hand, have enabled STEM-EEL characterization of plasmonic (*10*, *11*), nanophotonic (*12*, *13*), and phononic (*14*–*16*) systems in the low-loss (≲10 eV) regime with nanometer-scale spatial resolution. At these low energies (*17*, *18*), the STEM-EEL observable primarily probes the component of the generalized electromagnetic density of optical states (EMDOS) of the target specimen projected along the TEM axis (*19*, *20*). However, following the demonstration of vortex electron beams carrying quantized orbital angular momentum (OAM) (*21*, *22*), there has been considerable interest in studying OAM transfer between vortex free-electron states and atomic (*23*, *24*) and nanophotonic (*25*–*27*) targets. In particular, it was demonstrated that the symmetries of excited plasmonic modes could be controlled by pre- and post-selection of the transverse wave functions of the probing free electrons (*28*, *29*). More recently, a quasistatic theory was presented in which the transition dipole ${\hat{d}}_{fi}^{⊥}$ arising during transitions between spatially localized phase-shaped transverse electron states plays the role of an optical polarization analog (OPA) in EEL processes, allowing access to additional components of the target’s generalized EMLDOS (*30*). Despite the coexistence of the ${\hat{d}}_{fi}^{⊥}$ OPA in low-loss STEM-EEL measurements and the ${\hat{q}}_{⊥}$ OPA in core-loss scattering processes, the connection between these two OPAs has yet to be made explicit.

Here, we present a general theoretical framework for describing fully retarded inelastic electron scattering and elucidate the notion of and relationships between OPAs in these measurements. Using a formalism that explicitly accounts for the swift electron transverse degrees of freedom, we uncover an underlying connection between the two ostensibly distinct OPAs previously identified in LM- and OAM-resolved measurements under wide-field and focused beam conditions. Despite their apparent differences and regimes of applicability, the ${\hat{q}}_{⊥}$ and ${\hat{d}}_{fi}^{⊥}$ OPAs arising during LM and OAM transfer processes are both manifestations of the transverse components of the transition current density arising in our current-current response formalism. Numerical calculations highlighting the utility of phase-shaped EEL nanospectroscopy for determining mode symmetries and probing the three-dimensional (3D) polarization-resolved response field of a plasmonic dimer target with nanoscale spatial resolution are presented.

## RESULTS

**Fig. 1.**
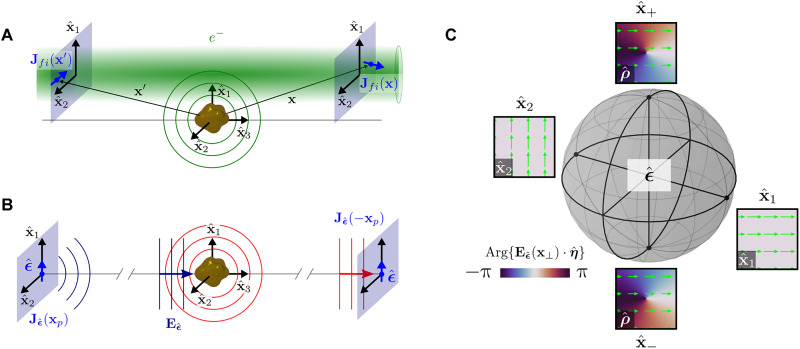
EEL and optical extinction processes. (**A**) Scheme showing the EEL process within the current-current response picture. (**B**) Scheme depicting the optical plane wave extinction process within the current-current response picture. Electromagnetic radiation sourced by a point dipole current density $J_{\hat{\epsilon }}$ located at position **x***_p_* and polarized along $\hat{\epsilon }$ interacts via the target with a mirrored observer dipole at −**x***_p_*. (**C**) Free-space optical polarization states $\hat{\epsilon }$ represented on the Poincaré sphere. Underlying color maps represent the transverse phase profiles $E_{\hat{\epsilon }}(x_{⊥},x_{3}=0)\cdot \hat{\text{η}}$, where $\hat{\text{η}}$ is a unit vector within the **x**_⊥_ plane.

Within the first Born approximation, the rate of scattering from the initial light-matter state ∣*i*⟩∣0⟩, describing initial electron state ∣*i*⟩ and target ground state ∣0⟩, to a given final state ∣*f*⟩∣*n*⟩ is equal to that found using Fermi’s golden rule with the interaction potential $\hat{V}=(e/2mc)(\hat{A}\cdot \hat{p}+\hat{p}\cdot \hat{A})$ in the generalized Coulomb gauge (*31*) defined by ∇ · [ɛ(**x**)**A**(**x**, *t*)] = 0 with zero scalar potential. The electron charge is −*e* and Gaussian units are used throughout this work. Using a mode expansion of the target’s vector potential $A(x,t)=\sum_{n}{\hat{a}}_{n}^{†}A_{n}^{\left(-\right)}(x)e^{+i\omega_{n}t}+{\hat{a}}_{n}A_{n}^{\left(+\right)}(x)e^{-i\omega_{n}t}$, the state-to-state frequency-resolved EEL transition rate becomes (see Materials and Methods)

wfiloss(ω)=2πℏ2∑n|1c∫dxAn(−)(x)⋅Jfi(x)|2δ(ω−ωif)δ(ω−ωn)=−8πℏ∫dxdx′Im{Jfi∗(x)⋅G(x,x′,ω)⋅Jfi(x′)}δ(ω−ωif)=4π2ℏω∫dxdx′Jfi∗(x)⋅ϱ(x,x′,ω)⋅Jfi(x′)δ(ω−ωif) (1)

where *E_if_* = ℏω*_if_* is the energy difference between initial ∣*i*⟩ and final ∣*f*⟩ electron states, **ϱ**(**x**, **x**′, ω) = (−2ω/π) Im {**G**(**x**, **x**′, ω)} is the generalized EMDOS tensor, and **G**(**x**, **x**′, ω) is the induced vector Helmholtz Green’s tensor. The free-electron transition current density

$$J_{fi}(x)=\frac{i\hbar e}{2m}\{\psi_{f}^{*}(x)\nabla \psi_{i}(x)-\psi_{i}(x)\nabla \psi_{f}^{*}(x)\}$$ (2)

is fully determined by the initial and final electron states, and its orientation determines which components of the generalized EMDOS tensor contribute to $w_{fi}^{\mathrm{loss}}(\omega )$ at each point in space (Fig. 1A). Meanwhile, the optical extinction cross section is often presented as σext(ω)=4π(ω/c)Im{ϵ^∗⋅α(ω)⋅ϵ^}, where **α**(ω) is the polarizability tensor characterizing the response of the target located at **x***_t_* to plane wave excitation with polarization unit vector $\hat{\epsilon }$. The optical cross section can be alternatively expressed in a form very similar to Eq. 1, e.g., (see Materials and Methods)

σext(ω)=4πωc1∣Eϵ^(xt,ω)∣2Im{−∫dxdx′Jϵ^∗(x,ω)⋅G(x,x′,ω)⋅Jϵ^(x′,ω)} (3)

by imagining that the incident plane wave field $E_{\hat{\epsilon }}$ is sourced by a point dipole with amplitude *p* located far away from the target at position **x***_p_* and characterized by current density $J_{\hat{\epsilon }}(x,\omega )=-i\omega p\delta (x-x_{p})\hat{\epsilon }$ as shown in Fig. 1B. Here, ${\hat{J}}_{\hat{\epsilon }}$ is locked to $\hat{\epsilon }$, which is in principle any arbitrary free photon pure polarization state described by a point on the Poincaré sphere (Fig. 1C), where antipodal points $\left\{{\hat{\epsilon }}_{1},{\hat{\epsilon }}_{2}\right\}$ and $\left\{{\hat{\epsilon }}_{+},{\hat{\epsilon }}_{-}\right\}$ describe linearly and circularly polarized light, respectively. Spatial maps of the plane wave electric field $E_{\hat{\epsilon }}(x_{⊥},x_{3}=0)$ (green arrows) with wave vector along ${\hat{x}}_{3}$ are shown for each of the four antipodal points indicated. Underlying color maps show the transverse phase profiles $E_{\hat{\epsilon }}(x_{⊥},x_{3}=0)\cdot \hat{\text{η}}$, where $\hat{\text{η}}$ is a unit vector within the **x**_⊥_ plane and is noted within each plot.

The connection between $\hat{\epsilon }$ in optical measurements and ${\hat{J}}_{fi}^{⊥}$ in EELS is determined by the identities of the initial and final free-electron states. If the electron wave function can be separated within an orthogonal coordinate system with ***x*** = (*x*_1_, *x*_2_, *x*_3_) and translational invariance along *x*_3_, then the wave function can be written as ψ(**x**) = Ψ(**x**_⊥_)*e*^*ik*_3_*x*_3_^ = Ψ_1_(*x*_1_)Ψ_2_(*x*_2_)*e*^*ik*_3_*x*_3_^, and the transition current density decomposes into transverse and longitudinal parts $J_{fi}(x)=[J_{fi}^{⊥}(x_{⊥})+J_{fi}^{∥}(x_{⊥}){\hat{x}}_{3}]e^{iq_{∥}x_{3}}$, where $q_{∥}=k_{3}^{i}-k_{3}^{f}$ is the longitudinal momentum transfer. These conditions on ψ(**x**) may be satisfied within the Cartesian as well as polar, elliptical, and parabolic cylindrical coordinate systems (*32*). Given the ability to perform EEL measurements with pre- and post-selection of free-electron transverse states with rationally sculpted phase profiles, full control may be exerted over the characteristics of $J_{fi}^{⊥}(x)$, permitting the construction of conventional OPAs and other more exotic structured light analogs (*33*), including radially and azimuthally polarized $J_{fi}^{⊥}(x)$ (Supplementary Materials). In particular, OPAs can be defined by identifying pairs of initial and final states such that ${\hat{J}}_{fi}^{⊥}(x_{⊥})\to {\hat{J}}_{fi}^{⊥}$ is position independent and described by a point on the Poincaré sphere shown in Fig. 1C with antipodal points constructed from unit vectors $\hat{x}$_1_ and $\hat{x}$_2_. To do so, we note that **J***_fi_*(**x**) · $\hat{x}$*_j_* (*j* = 1,2) vanishes provided that (i) Ψ*_j_*(*x_j_*) remains unchanged during interaction with the target and that (ii) Arg{Ψ*_j_*(*x_j_*)} is constant. Necessary conditions and a general OPA construction are discussed more fully in Materials and Methods.

**Fig. 2.**
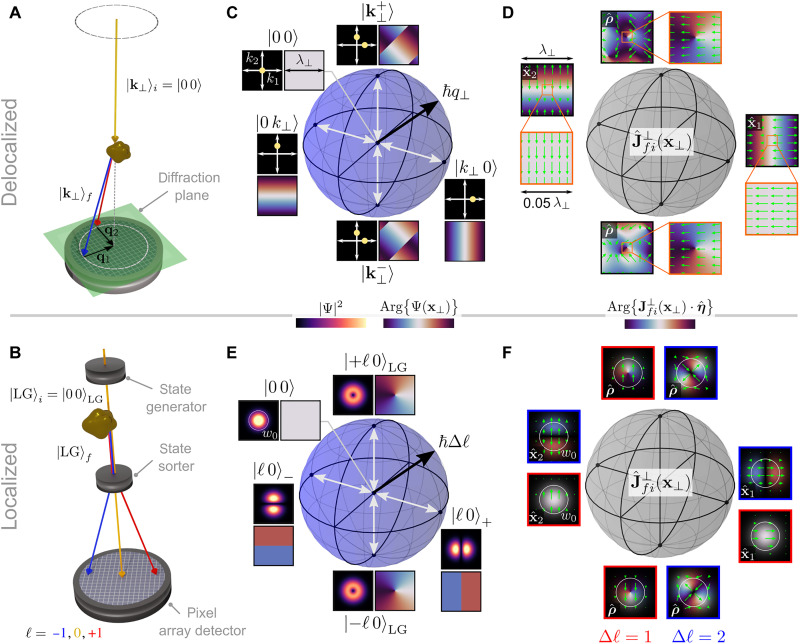
OPAs in phase-shaped EELS. Schemes showing (**A**) plane wave ∣00⟩ → ∣**k**_⊥_⟩*_f_* LM-resolved and (**B**) Laguerre-Gauss (LG) ∣00⟩_LG_ → ∣LG⟩*_f_* OAM-resolved EEL measurements. (**C**) Schematic representation of transitions between the ∣00⟩ transverse plane wave state with constant phase (sphere center) and other states with transverse wave vector magnitude *k*_⊥_ and nonuniform phase arranged around the Poincaré sphere. Reciprocal space probability densities and real-space transverse phase profiles over one transverse wavelength λ_⊥_ = 2π/∣**q**_⊥_∣ are shown for the states at the four antipodal points indicated. (**D**) Spatial maps of the transition current density $J_{fi}^{⊥}(x_{⊥},x_{3}=0)$ (green arrows) for transverse state transitions represented by white arrows in (C). Underlying color maps represent the phase profiles of $J_{fi}^{⊥}(x_{⊥})\cdot \hat{\text{η}}$, where $\hat{\text{η}}$ is noted within each plot. (**E**) Schematic representation of transitions between the ∣∣00⟩_LG_ state and superposition states with ℏℓ units of OAM arranged around the Poincaré sphere. Real-space probability density and transverse phase profiles are shown for the states at the four antipodal states indicated for 𝓁 = 1. The radius of the white circle is equal to the beam waist *w*_0_. (**F**) $J_{fi}^{⊥}(x_{⊥},x_{3}=0)$ (green arrows) for transverse state transitions represented by white arrows in (E) for Δ𝓁 = 1 (red) and Δ𝓁 = 2 (blue). Underlying color maps represent the phase profiles of $J_{fi}^{⊥}(x_{⊥})\cdot \hat{\text{η}}$.

The requisite pre- and post-selection transverse phase measurements underlying the OPA construction presented here are currently achievable; LM- and OAM-resolved EEL processes, shown schematically in Fig. 2 (A and B, respectively) constitute two well-known examples of these measurements. In its simplest realization, the LM-resolved measurement involves the preparation of an initial plane wave state ∣**k***_i_*⟩, which evolves into a different superposition of plane wave states via interaction with the target specimen. Post-selection of the final transverse state ∣**k***_f_*⟩ fixes the LM transfer ℏ**q** = ℏ(**k***_i_* − **k***_f_*) and can be accomplished via spatial filtering within the diffraction plane (*2*, *34*). Similarly, Fig. 2B depicts OAM-resolved EEL processes, where OAM state generators (*35*–*38*) and sorters (*39*, *40*) perform selection of initial and final vortex states with well-defined OAM. An expanded discussion of experimental considerations concerning phase-shaped EELS measurements is provided in the Supplementary Materials.

Consider, for example, transitions between electron plane wave states ∣**k**⟩ = ∣**k**_⊥_⟩ ∣*k*_3_⟩, ∣**k**_⊥_⟩ = ∣*k*_1_ *k*_2_⟩ denotes the transverse LM state with ∣00⟩ defining the plane wave oriented along the TEM axis. Figure 2C shows a Poincaré sphere with the surface composed of all transverse plane wave states with fixed transverse wave vector magnitude *k*_⊥_, and the ∣00⟩ state characterized by constant spatial phase profile at the sphere center. For any transitions between the ∣00⟩ state and a state on the sphere surface, the transverse LM transfer ℏ**q**_⊥_ = ℏ**k**_⊥_ fixes the radius of the sphere. Spanning the equatorial plane are the ∣*k*_⊥_0⟩ and ∣0*k*_⊥_⟩ states characterized by phase profiles independent of *x*_2_ and *x*_1_, respectively. The superposition states $|k_{⊥}^{\pm }\rangle =(1/\sqrt{2})[|k_{⊥}0\rangle \pm i|0k_{⊥}\rangle ]$ are located at the vertical pair of antipodal points. The reciprocal space wave function density and real space transverse phase are shown over one transverse wavelength λ_⊥_ = 2π/∣**q**_⊥_∣ for the four antipodal points indicated.

Although ∣00⟩ → ∣00⟩ transitions lead to ${\hat{J}}_{fi}(x)$ purely along $\hat{x}$_3_, **J***_fi_*(**x**) = (−ℏ*e*/2*mL*^3^)(2**k***_i_* − **q**)*e*^*i***q**·**x**^ if the final state consists of a single plane wave component with wave vector **k***_f_*, where $J_{fi}^{⊥}(x)\propto q_{⊥}e^{iq\cdot x}$ and *L* is the box quantization length. Figure 2D presents $J_{fi}^{⊥}(x_{⊥},x_{3}=0)$ for transitions between the ∣00⟩ state and the four antipodal states on the surface of the sphere in Fig. 2C (marked as white arrows). The spatial periodicity of the plane wave wave functions is inherited by $J_{fi}^{⊥}(x_{⊥})$, leading to spatial variation of the sign of the ${\hat{x}}_{1}$ and ${\hat{x}}_{2}$ components of $J_{fi}^{⊥}(x_{⊥})$ at the equatorial antipodal points, as well as sign and orientation variation at the vertically oriented antipodal points. However, when ∣**q**_⊥_∣ *d* ≪ 1, where *d* is the characteristic transverse length scale of the target, the magnified regions in Fig. 2D show that ${\hat{J}}_{fi}^{⊥}(x)$ becomes approximately independent of position and is oriented along ${\hat{q}}_{⊥}$ in the vicinity of the target. Thus, the resulting ${\hat{J}}_{fi}^{⊥}\approx {\hat{q}}_{⊥}$ Poincaré sphere becomes equivalent to that used to characterize optical plane wave polarization states in Fig. 1C. This is precisely the dipole limit discussed previously in the core-loss literature identifying ${\hat{q}}_{⊥}$ as the OPA in plane wave–based measurements (*4*–*6*, *8*).

Meanwhile, another Poincaré sphere can be constructed using the cylindrically symmetric and transversely localized Laguerre-Gauss (LG) ∣𝓁*p*⟩_LG_ transverse states (Fig. 2E), where ℏℓ is the OAM, *p* labels the number of radial nodes, and *w*_0_ is the beam waist. The ∣±𝓁0⟩_LG_ and superposition states ${|\ell 0\rangle }_{+}=(1/\sqrt{2})({|+\ell 0\rangle }_{\mathrm{LG}}+{|-\ell 0\rangle }_{\mathrm{LG}})$ and ${|\ell 0\rangle }_{-}=(1/i\sqrt{2})({|+\ell 0\rangle }_{\mathrm{LG}}-{|-\ell 0\rangle }_{\mathrm{LG}})$ are located at the vertical and equatorial antipodal points, respectively. As in Fig. 2C, the ∣00⟩_LG_ state with constant transverse phase is positioned at the sphere center such that transitions between the state at the center and states on the sphere surface are associated with transfers of Δ𝓁 units of OAM. The real space wave function densities and transverse phase profiles are presented for Δ𝓁 = 1. **J***_fi_*(**x**) acquires components transverse to ${\hat{x}}_{3}$ only when there is a transition between transverse free-electron states. Figure 2F shows $J_{fi}^{⊥}(x_{⊥})$ for transitions (marked as white arrows in Fig. 2E) between the uniform phase state at the sphere center and the four antipodal points shown on the surface for Δ𝓁 = 1 (red) and Δ𝓁 = 2 (blue). While the Δ𝓁 = 2 transition current densities have the symmetries required to excite quadrupolar target excitations, they do not constitute an OPA as defined here due to the spatial variation of ${\hat{J}}_{fi}^{⊥}(x_{⊥})$. This situation reflects the general relationship (*41*) between the LG OAM states and the Hermite-Gauss (HG) states, the latter of which are separable in the Cartesian coordinate system. Only in the particular case of Δ𝓁 = 1 are the equatorial antipodal states related to the first-order HG states, characterized by phase profiles independent of *y* and *x*, respectively, by ${|\ell =10\rangle }_{\pm }={|\begin{array}{c} 10\\ 01 \end{array}\rangle }_{\mathrm{HG}}$ (*30*). In this case, the orientation and spatial phase profiles of ${\hat{J}}_{fi}^{⊥}$ at the four Δ𝓁 = 1 antipodal points of Fig. 2F are identical to those associated with the electric field of circularly polarized light presented in Fig. 1C, satisfying the necessary OPA conditions.

Because of the delocalized (localized) nature of the plane wave (LG/HG) states, it is conventional to describe EEL measurements involving plane wave and LG/HG states in terms of the double differential scattering cross section ∂^2^σ/∂*E_if_*∂Ω and the state- and energy-resolved EEL probability Γ*_fi_* observables, respectively. Specializing to the Cartesian coordinate system with (*x*, *y*, *z*) = (**R**, *z*) and impact parameter **R** = **R**_0_, both observables are related to $w_{fi}^{\mathrm{loss}}(\omega )$ in Eq. 1 by (see Materials and Methods)

$$\left[\begin{array}{c} \frac{\partial^{2}\sigma }{\partial E_{if}\partial \text{Ω}}\\ {\text{Γ}}_{fi}(R_{0},\omega ) \end{array}\right]=\left[\begin{array}{c} -L^{6}{\left(\frac{2m}{4\pi \hbar^{2}}\right)}^{2}\left(\frac{k_{f}}{k_{i}}\right)\int \frac{d(\hbar \omega )}{2\pi }\\ \frac{mL^{2}}{\hbar^{2}k_{i}}\int \frac{dq_{∥}}{2\pi } \end{array}\right]\times w_{fi}^{\mathrm{loss}}(\omega )$$ (4)

where $\gamma_{i/f}=1/\sqrt{1-{\left(v_{i/f}/c\right)}^{2}}$, $k_{i/f}=\gamma_{i/f}mv_{i/f}/\hbar$, and $v_{i/f}$ are the initial and final electron speeds, and the nonrecoil approximation δ(ω − ω*_if_*) ≈ (1/*v_i_*)δ(*q*_||_ − ω/*v_i_*) is invoked in the lower expression. These observables are compared for a representative nanophotonic system composed of two 60 nm by 30 nm by 15 nm Ag rods with a 10-nm surface-to-surface gap along the dimer ($\hat{y}$) axis.

**Fig. 3.**
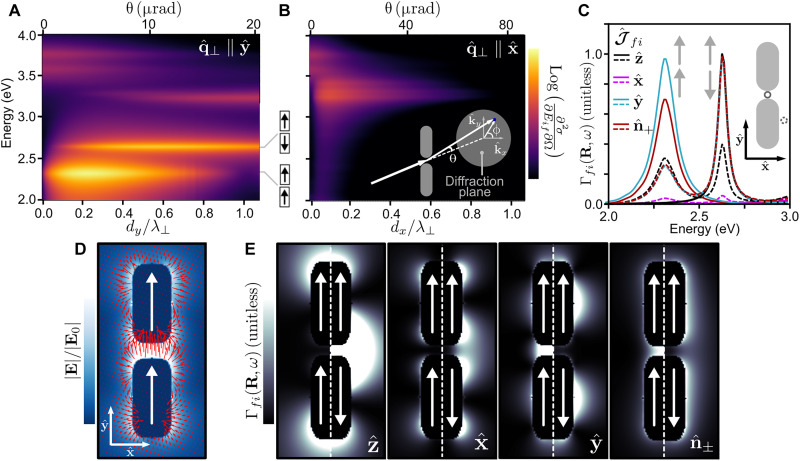
Phase-shaped EEL measurements of an Ag rod dimer system. (**A**) Double differential inelastic scattering cross section in the low-loss spectral region for scattering angles (θ, ϕ) = (0 − 20 μrad, π/2), such that $q_{⊥}=q_{⊥}\hat{y}$. (**B**) Same as (A) but for scattering angles (θ, ϕ) = (0 − 80 μrad, 0), such that $q_{⊥}=q_{⊥}\hat{x}$. (**C**) Γ*_fi_*(**R**, ω) within the spectral region containing the bonding (2.31 eV) and antibonding (2.64 eV) hybridized dipole modes for **R**_0_ on (solid) and off (dashed) the center of mass position. Trace colors denote ${\hat{J}}_{fi}$, where ${\hat{n}}_{\pm }=(1/\sqrt{2})(\hat{x}\pm i\hat{y})$. (**D**) Optically induced response field at the bonding mode energy for incident wave vector along $\hat{z}$ and polarization along $\hat{y}$. In-plane *xy* components of the vector field are shown in red. (**E**) Polarization-resolved spectrum images of bonding (left) and antibonding (right) modes for transitions between first-order LG/HG states and the Gaussian state. ${\hat{J}}_{fi}$ is labeled in each spectrum image.

Figure 3 (A and B) shows normalized ∂^2^σ/∂*E_if_*∂Ω spectra (log scale) for 200-keV electrons in the loss energy window containing the rods’ coupled surface plasmon modes, which were computed by adapting the electron-driven discrete dipole approximation (*e*-DDA) code (*42*, *43*) to evaluate the observables in Eq. 4 using Eqs. 1 and 2 as described in the Supplementary Materials. In Fig. 3A, the incoming electron plane wave is aligned along the TEM axis (θ, ϕ) = (0,0), and the outgoing plane waves emerge at angles (θ, ϕ) = (0 − 20 μrad, π/2) such that ${\hat{q}}_{⊥}$ is purely along $\hat{y}$. In this low-loss regime, the dipole limit with *d_y_* ≲ 0.05λ_⊥_ (Fig. 2C) is achieved for θ ≲ 1 μrad. As expected, the spectrum in Fig. 3A for θ = 1 μrad is dominated by the bonding dipole mode at 2.31 eV, which is accessible by optical plane wave excitation polarized along ${\hat{q}}_{⊥}=\hat{y}$ (see fig. S1). A direct comparison is presented in fig. S2 of the double differential inelastic electron scattering and optical extinction cross-section spectra for small electron scattering angles. As the detection angle increases in Fig. 3A, λ_⊥_ decreases and higher-order excitations such as the $\hat{y}$-oriented antibonding mode at 2.64 eV grow into the spectra (*44*). Figure 3B presents *∂*^2^σ/*∂E_if_∂*Ω spectra for detection angles (θ, ϕ) = (0 − 80 μrad,0), such that ${\hat{q}}_{⊥}$ is along $\hat{x}$. Besides the longitudinally oriented modes above 3.5 eV that dominate at θ = 0 in panels (A) and (B), the modes near 3.25 eV in the optical extinction spectra under $\hat{x}$ polarization appear for **q***_x_* ≠ 0 (see fig. S1). A clear connection between optical σ_ext_(ω) and *∂^2^σ/∂E_if_*∂Ω exists at small detection angles consistent with the dipole limit (Fig. 2D). The required angular resolution (≲1 μrad in the present case) is currently achievable experimentally (*34*, *36*, *45*, *46*).

In the case of localized LG state transitions (Fig. 2E), Γ*_fi_* can be put into the form

Γfi(R0,ω)=−1πℏ2ωRe{∫dRdzJfi∗(R,z,ω)⋅Efi(R,z,ω)} (5)

by introducing the induced field **E***_fi_*(**x**, ω) = −4π*i*ω∫*d***x**′**G**(**x**, **x**′, ω) · **J***_fi_*(**x**′, ω) sourced by the transition current density **J***_fi_*(**x**, ω) = (*L*/*v*_i_)**J***_fi_*(**x**) in the presence of the target, with ω = *q*_||_*v_i_* within the nonrecoil approximation as explained in Materials and Methods. When *w*_0_ is small, such that **E***_fi_*(**x**, ω) ≈ **E***_fi_*(**R**_0_, *z*, ω)

Γfi(R0,ω)=−1πℏ2ωRe{Jfi∗⋅∫dzEfi(R0,z,ω)e−iωz/vi} (6)

where $J_{fi}=\int dR\;J_{fi}(x,\omega )\;e^{-i\omega z/v_{i}}=(-e/mv_{i})\{\langle {\text{Ψ}}_{f}|{\hat{p}}_{⊥}|{\text{Ψ}}_{i}\rangle +[(2p_{i}-\hbar q_{∥})/2]\langle {\text{Ψ}}_{f}|{\text{Ψ}}_{i}\rangle \hat{z}\}.$ Therefore, in the narrow beam limit, the observable Γ*_fi_*(**R**_0_, ω) is proportional to the real part of the Fourier expansion coefficient of the target response field for the particular plane wave component propagating along the TEM axis with wave vector magnitude equal to the longitudinal transfer momentum *q*_||_ = ω/*v_i_* and polarization direction ${\hat{J}}_{fi}^{*}$. In this limit, ${\hat{J}}_{fi}^{⊥}=0$ unless Δ𝓁 = ±1, in which case ${\hat{J}}_{fi}^{⊥}={\hat{J}}_{fi}^{⊥}={\hat{d}}_{fi}^{⊥}$ (see the Supplementary Materials) as shown in Fig. 2F. Figure 3C presents Γ*_fi_*(**R**, ω) evaluated numerically with the *e*-DDA code (*42*, *43*, *47*) as discussed in the Supplementary Materials using Eq. 6 for ${\hat{J}}_{fi}^{⊥}$ at the antipodal points in Fig. 2F and ${\hat{J}}_{fi}^{⊥}∥\hat{z}$ for the transition ∣00⟩_LG_ → ∣00⟩_LG_ within the spectral region containing the bonding and antibonding hybridized dipole modes. Trace colors denote ${\hat{J}}_{fi}$, while solid and dashed lines indicate **R**_0_ positioned at, or displaced from, respectively, the dimer center of mass. At the center of mass position, a conventional EEL process with no transition between transverse state (solid black) does (does not) couple to the optically dark (bright) antibonding (bonding) mode. In contrast, when ${\hat{J}}_{fi}={\hat{J}}_{fi}^{⊥}$, the loss functions exhibit peaks at the bonding, but not antibonding, mode. When **R**_0_ is displaced to a position of lower symmetry, all electron scattering processes considered couple to both bonding and antibonding modes.

Polarization-resolved spectrum images can be collected via hyperspectral imaging by scanning **R**_0_ over the target specimen. Although Γ*_fi_*(**R**, ω) is nonlocal in the *z* direction (*19*, *20*), conventional (${\hat{J}}_{fi}=\hat{z}$) and polarization-resolved (${\hat{J}}_{fi}={\hat{J}}_{fi}^{⊥}$) spectrum images can often be rationalized by considering the electric field (Fig. 3D). Figure 3E presents polarization-resolved spectrum images for loss energies matching the bonding (left) and antibonding (right) mode energy and for transitions between electron transverse states indicated by the transition current unit vector ${\hat{J}}_{fi}$ labeled in each image. It is evident upon comparison of the spectrum images at the bonding mode energy and Fig. 3D that the regions of space, where Γ*_fi_*(**R**, ω) is large, closely track positions where $E\cdot {\hat{J}}_{fi}$ is appreciable. The polarization-resolved spectrum images at the antibonding energy exhibit the expected nodal behavior at the origin for each ${\hat{J}}_{fi}$. This example demonstrates the additional wealth of information that can be accessed using OPAs in phase-shaped EEL measurements, highlighting commonalities and differences arising for pre- and post-selection of transverse plane wave and LG/HG states.

## DISCUSSION

Owing to recently developed experimental techniques for manipulating free-electron wave functions, inelastic electron scattering between selected phase-shaped transverse states is emerging as a powerful addition to the rapidly developing nanoscale imaging toolset. By using a fully quantum mechanical treatment that explicitly accounts for the transverse electron degrees of freedom with fully retarded light-matter interactions, we show that the transition current density $J_{fi}^{⊥}$ plays the role of OPA in EEL measurements and provides a general prescription for constructing OPAs that mimic the polarization properties of free-space optical plane waves. The insight gained from the perspective suggested by our approach is used to demonstrate an underlying connection between the two ostensibly distinct OPAs previously identified in the core- and low-loss regimes under wide-field and focused beam conditions, respectively, and discuss the conditions required to most closely approximate ideal OPAs. Example calculations for a plasmonic rod dimer are presented to highlight the utility of phase-shaped EEL nanospectroscopy for determining mode symmetries and for probing the 3D polarization-resolved response field of a target with nanoscale spatial resolution. Although the primary focus is placed on developing free space photon OPAs, the procedure outlined for constructing ${\hat{J}}_{fi}^{⊥}$ along arbitrary curvilinear coordinate directions is general and can be applied to generate more exotic structured light (*33*) analogs that can couple to other desired target mode symmetries. The fully retarded formalism used here is consistent with that used to describe laser-stimulated phase-shaped electron energy gain measurements (*48*), setting the stage for time-resolved phase-shaped measurements in ultrafast TEMs (*18*, *49*). The ability to perform high-fidelity pre- and post-selection of the free-electron state, as well as understanding the role of transverse state coherence and its ability to be manipulated, in conventional and ultrafast TEM experiments remains a current area of interest to experiment and theory. These techniques complement other methods with state-of-the-art time and space resolution such as interferometric time-resolved photoemission electron microscopy (*50*), which has been used to image nanoscale spin and field textures with nontrivial topology (*51*).

## MATERIALS AND METHODS

### State- and frequency-resolved EEL rate

Within the first Born approximation, the rate at which a free electron prepared in initial state ∣*i*⟩ transitions to a final state ∣*f*⟩, while simultaneously depositing a single excitation into the 𝓁th target mode (i.e., ∣0_𝓁_⟩ → ∣1_𝓁_⟩), is given by Fermi’s golden rule

$$w_{fi}^{\mathrm{loss}}=\frac{2\pi }{\hbar }{|\langle f|\langle n_{\ell }|\hat{V}|0_{\ell }\rangle |i\rangle |}^{2}\delta (E_{f}^{\mathrm{total}}-E_{i}^{\mathrm{total}})$$ (7)

Using the identity $\delta (E_{f}^{\mathrm{total}}-E_{i}^{\mathrm{total}})=\hbar^{-1}\int d\omega \;\delta (\omega -\omega_{if})\delta (\omega -\omega_{\ell })$, where ℏω*_if_* is the energy loss of the free electron and ℏω_𝓁_ is the energy of the excited target mode, the frequency-resolved transition rate can be expressed as

$$w_{fi}^{\mathrm{loss}}(\omega )=\frac{2\pi }{\hbar^{2}}{|\langle f|\langle 1_{\ell }|\hat{V}|0_{\ell }\rangle |i\rangle |}^{2}\delta (\omega -\omega_{if})\delta (\omega -\omega_{\ell })$$ (8)

Working in the generalized Coulomb gauge (*31*) defined by ∇ · [ɛ(**x**)**A**(**x**, *t*)] = 0 with zero scalar potential, the interaction potential is $\hat{V}=(e/2mc)(\hat{A}\cdot \hat{p}+\hat{p}\cdot \hat{A})$, and the matrix element for an arbitrary electron transition (∣*i*⟩ → ∣*f*⟩) can be written in terms of the transition current density **J***_fi_* defined in Eq. 2. Explicitly,

$$\begin{array}{rl} \left\langle f|\hat{V}|i\right\rangle & =\frac{e}{2mc}\int dx\;dx^{\prime }\;\langle f|x^{\prime }\rangle \langle x^{\prime }|\hat{A}\cdot \hat{p}+\hat{p}\cdot \hat{A}|x\rangle \langle x|i\rangle \\ & =\frac{-i\hbar e}{2mc}\int dx\;\hat{A}(x)\cdot \psi_{f}^{*}(x)\nabla \psi_{i}(x)\\ & \;-\frac{i\hbar e}{2mc}\int dx\;\{\psi_{f}^{*}(x)\psi_{i}(x)[\nabla \cdot \hat{A}(x)]+\hat{A}(x)\cdot \psi_{f}^{*}(x)\nabla \psi_{i}(x)\}\\ & =\frac{-2i\hbar e}{2mc}dx\;\hat{A}(x)\cdot \psi_{f}^{*}(x)\nabla \psi_{i}(x)\\ & \;-\frac{i\hbar e}{2mc}\int dx\;\psi_{f}^{*}(x)\psi_{i}(x)[\nabla \cdot \hat{A}(x)]\\ & =\frac{-2i\hbar e}{2mc}\int dx\;\hat{A}(x)\cdot \psi_{f}^{*}(x)\nabla \psi_{i}(x)\\ & \;+\frac{i\hbar e}{2mc}\int dx\;\hat{A}(x)\cdot \{\nabla \psi_{f}^{*}(x)\psi_{i}(x)+\psi_{f}^{*}(x)\nabla \psi_{i}(x)\}\\ & =\frac{-1}{c}\int dx\;\hat{A}(x)\cdot \frac{i\hbar e}{2m}\{\psi_{f}^{*}(x)\nabla \psi_{i}(x)-\psi_{i}(x)\nabla \psi_{f}^{*}(x)\}\\ & =-\frac{1}{c}\int dx\;\hat{A}(x)\cdot J_{fi}(x) \end{array}$$ (9)

In going from the third to fourth lines of Eq. 9, the surface term generated by integrating by parts has been assumed to vanish. The total transition rate associated with EEL ℏω*_if_* is found by summing over all possible final excited states of the target, which are denoted by 𝓁 in Eq. 8. This is facilitated by expanding the vector potential as $A(x,t)=\sum_{n}{\hat{a}}_{n}^{†}A_{n}^{\left(-\right)}(x)e^{+i\omega_{n}t}+{\hat{a}}_{n}A_{n}^{\left(+\right)}(x)e^{-i\omega_{n}t},$ where $A_{n}^{\left(+\right)}(x)=c\sqrt{2\pi \hbar /\omega_{n}}\;f_{n}(x)$ and $A_{n}^{\left(-\right)}(x)=c\sqrt{2\pi \hbar /\omega_{n}}\;f_{n}^{*}(x)$ are the positive and negative frequency components of the vector potential written in terms of spatial mode functions **f***_n_*(**x**), which satisfy the generalized Helmholtz equation. Because we are considering only the EEL, $\sum_{\ell }\langle 1_{\ell }|\hat{A}(x^{\prime },t)|0_{\ell }\rangle$ $=\sum_{n\ell }c\sqrt{2\pi \hbar /\omega_{\ell }}\;f_{\ell }^{*}(x^{\prime })e^{i\omega_{\ell }t}$ $\delta_{n\ell }=\sum_{n}A_{n}^{\left(-\right)}(x^{\prime })$ and consequently for the conjugate process, $\sum_{\ell }\langle 0_{\ell }|\hat{A}(x,t)|1_{\ell }\rangle^{*}=\sum_{n\ell }c\sqrt{2\pi \hbar /\omega_{\ell }}\;f_{\ell }(x)e^{-i\omega_{\ell }t}\delta_{n\ell }=\sum_{n}A_{n}^{\left(+\right)}(x).$ Inserting these matrix elements into Eq. 8 yields main text Eq. 1, i.e.,

wfiloss(ω)=∑ℓ2πc2ℏ2|⟨1ℓ∣∫dxA^(x)⋅Jfi(x)∣0ℓ⟩|2δ(ω−ωif)δ(ω−ωℓ)=∑n2πc2ℏ2∫dxdx′[An(+)(x)⋅Jfi∗(x)][An(−)(x)⋅Jfi(x′)]×δ(ω−ωif)δ(ω−ωn)=4π2ℏ∫dxdx′Jfi∗(x)⋅[∑n1ωnfn(x)fn∗(x′)δ(ω−ωn)]⋅Jfi(x′)δ(ω−ωif)=4π2ℏ∫dxdx′Jfi∗(x)⋅[−2πIm{G(x,x′,ω)}]⋅Jfi(x′)δ(ω−ωif)=4π2ℏω∫dxdx′Jfi∗(x)⋅ϱ(x,x′,ω)⋅Jfi(x′)δ(ω−ωif) (11)

Because of the reciprocity property of the Green’s dyadic **G**(**x**_1_, **x**_2_, ω) = **G**^**T**^(**x**_2_, **x**_1_, ω),

∫dxdx′Jfi∗(x)⋅Im{G(x,x′,ω)}⋅Jfi(x′)=∫dxdx′Im{Jfi∗(x)⋅G(x,x′,ω)⋅Jfi(x′)} (12)

and Eq. 10 can be alternatively expressed as

wfiloss(ω)=4π2ℏ∫dxdx′Jfi∗(x)⋅[−2πIm{G(x,x′,ω)}]⋅Jfi(x′)δ(ω−ωif)=−8πℏ∫dxdx′Im{Jfi∗(x)⋅G(x,x′,ω)⋅Jfi(x′)}δ(ω−ωif) (13)

### Optical extinction cross section

The optical plane wave extinction cross section for an isolated dipolar target is

σext(ω)=4πωcIm{ϵ^∗⋅α(ω)⋅ϵ^} (14)

where **α**(ω) is the dipole polarizability tensor characterizing the target response to plane wave excitation with polarization unit vector $\hat{\epsilon }$. This expression can alternatively be expressed as

σext(ω)=4πωc1∣Eϵ^(xt,ω)∣2Im{Eϵ^∗(xt,ω)⋅α(ω)⋅Eϵ^(xt,ω} (15)

where $E_{\hat{\epsilon }}(x_{t})$ is the plane wave electric field at the position of the target **x***_t_*. A plane wave with polarization $\hat{\epsilon }$ directed along $\hat{k}$ can be viewed as though sourced by a point dipole at position **x***_p_* with current density $J_{\hat{\epsilon }}(x,\omega )=-i\omega p\delta (x-x_{p})\hat{\epsilon }$ and $\hat{k}=-{\hat{x}}_{p}$ (*18*), because

$$\begin{array}{cc} E_{\hat{\epsilon }}(x,\omega ) & =-4\pi i\omega \int dx^{\prime }G_{0}(x,x^{\prime },\omega )\cdot J_{\hat{\epsilon }}(x^{\prime },\omega )\\ & =[k^{2}I+\nabla \nabla ]\frac{e^{ik|x-x_{p}|}}{|x-x_{p}|}\cdot p\hat{\epsilon } \end{array}$$ (16)

where $J_{\hat{\epsilon }}$ and $E_{\hat{\epsilon }}$ are understood to oscillate harmonically as *e*^−*i*ω*t*^ and the free space Green dyadic is

$$G_{0}(x,x^{\prime },\omega )=-\frac{1}{4\pi \omega^{2}}[k^{2}I+\nabla \nabla ]\frac{e^{ik|x-x\prime |}}{|x-x^{\prime }|}$$ (17)

Taking the source dipole position **x***_p_* to be a large distance from the target and using the fact that $\hat{k}=-{\hat{x}}_{p}$

$$G_{0}(x,x^{\prime },\omega )\to -\frac{k^{2}}{4\pi \omega^{2}}\frac{e^{ik|x-x\prime |}}{|x-x^{\prime }|}I$$ (18)

and

$$E_{\hat{\text{ϵ}}}(x,\omega )\to \frac{i}{\omega }\int dx^{\prime }k^{2}\frac{e^{ik|x-x^{\prime }|}}{|x-x^{\prime }|}I\cdot J_{\hat{\text{ϵ}}}(x^{\prime },\omega )$$ (19)

which establishes that $E_{\hat{\epsilon }}∥J_{\hat{\epsilon }}∥\hat{\epsilon }$ as desired. The optical extinction cross section can therefore be written as

σext(ω)=4πωc1∣Eϵ^(xt,ω)∣2Im{∫dxdx′Jϵ^∗(x,ω)⋅(+4πiω)G0(x,xt)⋅α(ω)⋅(−4πiω)G0(xt,x′)⋅Jϵ^(x′,ω)}=−4πωc1∣Eϵ^(xt,ω)∣2Im{∫dxdx′Jϵ^∗(x,ω)⋅(+4πiω)G0(x,xt)⋅α(ω)⋅(−4πiω)G0(xt,x′)⋅Jϵ^(x′,ω)}=−4πωc1∣Eϵ^(xt,ω)∣2Im{∫dxdx′Jϵ^∗(x,ω)⋅G(x,x′,ω)⋅Jϵ^(x′,ω)} (20)

where

$$G(x,x^{\prime },\omega )=(+4\pi i\omega )G_{0}(x,x_{t})\cdot \text{α}(\omega )\cdot (-4\pi i\omega )G_{0}(x_{t},x^{\prime })$$ (21)

with the form of **G**_0_ defined in Eq. 18.

Target responses beyond the single dipole approximation can be evaluated using the discrete dipole approximation (*47*), whereby the continuous target is represented by a collection of dipoles interacting self-consistently under a driving field $E_{\hat{\epsilon }}$. In this case, **G**(**x**, **x**′, ω) can be expanded as (*25*, *30*)

$$\begin{array}{rl} G(x,x^{\prime },\omega ) & =\sum_{jj^{\prime }}(+4\pi i\omega )G_{0}(x,x_{j},\omega )\cdot {\left[{\text{α}}^{\left(-1\right)}+4{\pi \omega }^{2}G_{0}\right]}_{jj^{\prime }}^{\left(-1\right)}\\ & \;\cdot (-4\pi i\omega )G_{0}(x_{j^{\prime }}^{\prime },x^{\prime },\omega ) \end{array}$$ (22)

### Optical polarization analogs

Suppose that the free-electron wave function can be separated within an orthogonal coordinate system with variables *x*_1_, *x*_2_, *x*_3_, i.e., ψ(**x**) = Ψ_1_(*x*_1_)Ψ_2_(*x*_2_)Ψ_3_(*x*_3_), then the transverse transition current can be expressed as

$$\begin{array}{rl} J_{fi}^{⊥}(x) & =\frac{i\hbar e}{2m}\{{\text{Ψ}}_{⊥f}^{*}(x_{⊥})[\nabla_{⊥}{\text{Ψ}}_{⊥i}(x_{⊥})]-{\text{Ψ}}_{⊥i}(x_{⊥})[\nabla_{⊥}{\text{Ψ}}_{⊥f}^{*}(x_{⊥})]\}{\text{Ψ}}_{3f}^{*}(x_{3}){\text{Ψ}}_{3i}(x_{3})\\ & =\frac{i\hbar e}{2m}\{{\text{Ψ}}_{1f}^{*}(x_{1}){\text{Ψ}}_{2f}^{*}(x_{2})[\nabla_{⊥}{\text{Ψ}}_{1i}(x_{1}){\text{Ψ}}_{2i}(x_{2})]\\ & \;-{\text{Ψ}}_{1i}(x_{1}){\text{Ψ}}_{2i}(x_{2})[\nabla_{⊥}{\text{Ψ}}_{1f}^{*}(x_{1}){\text{Ψ}}_{2f}^{*}(x_{2})]\}{\text{Ψ}}_{3f}^{*}(x_{3}){\text{Ψ}}_{3i}(x_{3})\\ & =\frac{i\hbar e}{2m}\{{\text{Ψ}}_{1f}^{*}(x_{1}){\text{Ψ}}_{2f}^{*}(x_{2})\left[\frac{1}{h_{1}}\frac{\partial {\text{Ψ}}_{1i}(x_{1})}{\partial x_{1}}{\text{Ψ}}_{2i}(x_{2}){\hat{x}}_{1}+\frac{1}{h_{2}}{\text{Ψ}}_{1i}(x_{1})\frac{\partial {\text{Ψ}}_{2i}(x_{2})}{\partial x_{2}}{\hat{x}}_{2}\right]\\ & \;-{\text{Ψ}}_{1i}(x_{1}){\text{Ψ}}_{2i}(x_{2})\left[\frac{1}{h_{1}}\frac{\partial {\text{Ψ}}_{1f}^{*}(x_{1})}{\partial x_{1}}{\text{Ψ}}_{2f}^{*}(x_{2}){\hat{x}}_{1}+\frac{1}{h_{2}}{\text{Ψ}}_{1f}^{*}(x_{1})\frac{\partial {\text{Ψ}}_{2f}^{*}(x_{2})}{\partial x_{2}}{\hat{x}}_{2}\right]\}\\ & \;{\text{Ψ}}_{3f}^{*}(x_{3}){\text{Ψ}}_{3i}(x_{3})\\ & =\frac{i\hbar e}{2m}\{\frac{1}{h_{1}}{\text{Ψ}}_{2f}^{*}(x_{2}){\text{Ψ}}_{2i}(x_{2})\left[{\text{Ψ}}_{1f}^{*}(x_{1})\frac{\partial {\text{Ψ}}_{1i}(x_{1})}{\partial x_{1}}-{\text{Ψ}}_{1i}(x_{1})\frac{\partial {\text{Ψ}}_{1f}^{*}(x_{1})}{\partial x_{1}}\right]{\hat{x}}_{1}\\ & \;+\frac{1}{h_{2}}{\text{Ψ}}_{1f}^{*}(x_{1}){\text{Ψ}}_{1i}(x_{1})\left[{\text{Ψ}}_{2f}^{*}(x_{2})\frac{\partial {\text{Ψ}}_{2i}(x_{2})}{\partial x_{2}}-{\text{Ψ}}_{2i}(x_{2})\frac{\partial {\text{Ψ}}_{2f}^{*}(x_{2})}{\partial x_{2}}\right]{\hat{x}}_{2}\}\\ & \;{\text{Ψ}}_{3f}^{*}(x_{3}){\text{Ψ}}_{3i}(x_{3}) \end{array}$$ (23)

where *h*_μ_ is the scale factor associated with coordinate *x*_μ_. It is evident that the ${\hat{x}}_{\mu }$ component cof the transition current density vanishes when

$${\text{Ψ}}_{f\nu }^{*}{\text{Ψ}}_{i\nu }\left[{\text{Ψ}}_{f\mu }^{*}\frac{\partial {\text{Ψ}}_{i\mu }}{\partial x_{\mu }}-{\text{Ψ}}_{i\mu }\frac{\partial {\text{Ψ}}_{f\mu }^{*}}{\partial x_{\mu }}\right]=0$$ (24)

where μ, ν = 1,2 with μ ≠ ν. Provided ${\text{Ψ}}_{f\nu }^{*}{\text{Ψ}}_{i\nu }\neq 0$, and writing the wave function in polar form Ψ_μ_ = *A*(*x*_μ_)*e*^*i*ϕ(*x*_μ_)^ with *A* ∈ ℝ_>0_ and ϕ ∈ [0,2π], this condition can be rewritten as

$$A_{f}\left[\frac{\partial A_{i}}{\partial x_{\mu }}+i\phi_{i}A_{i}\frac{\partial \phi_{i}}{\partial x_{\mu }}\right]=A_{i}\left[\frac{\partial A_{f}}{\partial x_{\mu }}-i\phi_{f}A_{f}\frac{\partial \phi_{f}}{\partial x_{\mu }}\right]$$ (25)

If *f* = *i* and ϕ*_i_* = ϕ*_f_* = ϕ, then this becomes

$$\frac{\partial \phi }{\partial x_{\mu }}=-\frac{\partial \phi }{\partial x_{\mu }}$$ (26)

This condition is satisfied if the phase ϕ is strictly constant. In summary, the *x*_μ_ component of $J_{fi}^{⊥}$ vanishes provided that (i) Ψ_*i*μ_(*x*_μ_) = Ψ_*f*μ_(*x*_μ_) and that (ii) Arg{Ψ_μ_(*x*_μ_)} is constant.

OPAs can be defined, therefore, provided that pairs of initial Ψ_⊥*i*_ and final Ψ_⊥*f*_ free-electron transverse states can be identified, such that the conditions

$$\begin{array}{cc} {\text{Ψ}}_{f\nu }^{*}{\text{Ψ}}_{i\nu }\left[{\text{Ψ}}_{f\mu }^{*}\frac{\partial {\text{Ψ}}_{i\mu }}{\partial x_{\mu }}-{\text{Ψ}}_{i\mu }\frac{\partial {\text{Ψ}}_{f\mu }^{*}}{\partial x_{\mu }}\right] & =0\\ {\text{Ψ}}_{f\mu }^{*}{\text{Ψ}}_{i\mu }\left[{\text{Ψ}}_{f\nu }^{*}\frac{\partial {\text{Ψ}}_{i\nu }}{\partial x_{\nu }}-{\text{Ψ}}_{i\nu }\frac{\partial {\text{Ψ}}_{f\nu }^{*}}{\partial x_{\nu }}\right] & \neq 0 \end{array}$$ (27)

may be simultaneously satisfied. In these cases, a Poincaré sphere may be defined from ${\hat{J}}_{fi}^{⊥}\in \{{\hat{x}}_{1},{\hat{x}}_{2},{\hat{x}}_{\pm }=(1/\sqrt{2})({\hat{x}}_{1}\pm i{\hat{x}}_{2})\}$, and it is possible to realize ${\hat{J}}_{fi}^{⊥}$ at an arbitrary point on this Poincaré sphere using suitable coherent superpositions of the initial and final wave functions used to define the antipodal points. Two examples of such a Poincaré sphere construction within the Cartesian coordinate systems are presented in Fig. 2 (D and F), which involve transitions between wave functions constructed from states with well-defined LM (Fig. 2C) and OAM (Fig. 2E), respectively. The cases of radially $\hat{\text{ρ}}$ and azimuthally $\hat{\text{φ}}$ polarized ${\hat{J}}_{fi}^{⊥}$ are discussed in the Supplementary Materials and shown in fig. S3.

### Fully-retarded double differential inelastic scattering cross section

The total frequency-resolved inelastic scattering cross section σ(ω) can be defined by dividing the state- and frequency-resolved loss rate $w_{fi}^{\mathrm{loss}}(\omega )$ from Eq. 13 by the incoming plane wave particle flux ℏ*k_i_*/*mL*^3^ and summing over final electron states with ∑_**k***_f_*_ ‍ → (*L*/2π)^3^∫*d***k***_f_*, which gives

$$\begin{array}{cc} \sigma (\omega ) & =\frac{mL^{3}}{\hbar k_{i}}{\left(\frac{L}{2\pi }\right)}^{3}\int dk_{f}w_{fi}^{\mathrm{loss}}(\omega )\\ & =\frac{mL^{3}}{\hbar k_{i}}{\left(\frac{L}{2\pi }\right)}^{3}\int d\text{Ω}dk_{f}\;k_{f}^{2}w_{fi}^{\mathrm{loss}}(\omega ) \end{array}$$ (28)

The angular- and frequency-resolved cross section is then

$$\frac{\partial \sigma (\omega )}{\partial \text{Ω}}=\frac{mL^{3}}{\hbar k_{i}}{\left(\frac{L}{2\pi }\right)}^{3}\int dk_{f}\;k_{f}^{2}w_{fi}^{\mathrm{loss}}(\omega )$$ (29)

In addition to integrating out the explicit frequency dependence, the integral over the final wave vector magnitude can be expressed as an integral over loss energy *E_if_* due to the relativistic energy-momentum free-particle dispersion relation. Explicitly, *dE_if_* = −(*ℏ*^2^/*m*)*k_f_dk_f_* and

$$\frac{\partial \sigma }{\partial \text{Ω}}=-L^{6}{\left(\frac{2m}{4\pi \hbar^{2}}\right)}^{2}\int dE_{if}\;\left(\frac{k_{f}}{k_{i}}\right)\int \frac{d(\hbar \omega )}{2\pi }w_{fi}^{\mathrm{loss}}(\omega )$$ (30)

where $k_{i/f}=\gamma_{i/f}mv_{i/f}/\hbar$ and $\gamma_{i/f}=1/\sqrt{1-{\left(v_{i/f}/c\right)}^{2}}$. Consequently,

$$\begin{array}{rl} \frac{\partial^{2}\sigma }{\partial E_{if}\partial \text{Ω}} & =-L^{6}{\left(\frac{2m}{4\pi \hbar^{2}}\right)}^{2}\left(\frac{k_{f}}{k_{i}}\right)\int \frac{d(\hbar \omega )}{2\pi }\;w_{fi}^{\mathrm{loss}}(\omega )\\ & =-L^{6}{\left(\frac{2m}{4\pi \hbar^{2}}\right)}^{2}\frac{\hbar }{2\pi }\left(\frac{k_{f}}{k_{i}}\right)w_{fi}^{\mathrm{loss}}(\omega_{if}) \end{array}$$ (31)

which is identical to the double differential inelastic scattering cross section given in Eq. 4. Explicit analytic double differential cross-section expressions in the case of an isolated dipolar target, discussion of the quasistatic limit of this theory, and additional details concerning numerical evaluation of the double differential cross section are included in the Supplementary Materials.

### Fully retarded state- and energy-resolved EEL probability and the narrow beam limit

The energy-resolved loss probability is found by multiplying the frequency-resolved loss rate in Eq. 13 by the time required for the electron to traverse the quantization length (*L*/*v_i_*), summing over all the possible final electron momenta directed along the TEM axis (with $k_{f}^{z}$ || *q*_||_) and dividing by *ℏ*, giving

Γfi(R0,ω)=L2ℏvi∫dq∥2πwfiloss(ω)=−4viℏ2∫dq∥∫dxdx′Im{LviJfi∗(x)⋅G(x,x′,ω)⋅LviJfi(x′)}δ(ω−ωif) (32)

which is equivalent to the form given in Eq. 4. This state- and energy-resolved EEL probability can be re-expressed in terms of the induced electric field sourced by **J***_fi_* in the presence of the target using (*11*) with **J***_fi_*(x, ω) = (*L*/*v_i_*)**J***_fi_*(**x**). This yields

Γfi(R0,ω)=−4viℏ2∫dq∥Im{∫dxLviJfi∗(x)⋅(−14iπω)Efi(x,ω)}δ(ω−ωif)=−∫dq∥Re{viπℏ2ω∫dxLviJfi∗(x)⋅Efi(x,ω)}δ(ω−ωif) (33)

The integral over longitudinal momentum transfers can be performed trivially by invoking the standard nonrecoil approximation (*17*), whereby the energy associated with transverse recoils is neglected during the consideration of the trailing energy-conserving delta function in Eq. 33. The connection between ω*_if_* and *q*_||_ is found within the limit of *q*_||_ ≪ *k_i_*, *k_f_* as

$$\begin{array}{rl} E_{if}\equiv \hbar \omega_{if} & =\sqrt{{\left(mc^{2}\right)}^{2}+{\left(c\hbar k_{i}\right)}^{2}}-\sqrt{{\left(mc^{2}\right)}^{2}+{\left(c\hbar k_{f}\right)}^{2}}\\ & =\sqrt{{\left(mc^{2}\right)}^{2}+{\left(c\hbar k_{i}\right)}^{2}}-\sqrt{{\left(mc^{2}\right)}^{2}+{\left(c\hbar k_{i}\right)}^{2}}\\ & \times {\left[1+\frac{{\left(\hbar c\right)}^{2}\left(q_{∥}^{2}-2k_{i}q_{∥}\right)}{{\left(mc^{2}\right)}^{2}+{\left(c\hbar k_{i}\right)}^{2}}\right]}^{1/2}\\ & \approx \hbar q_{∥}v_{i} \end{array}$$ (34)

such that ω*_if_* ≈ *v_i_q*_||_.

As a result,

Γfi(R0,ω)=−1πℏ2ω∫dq∥Re{∫dRdzLviJfi∗(R,z)⋅Efi(R,z,ω)}δ(q∥−ω/vi)=−1πℏ2ωRe{∫dRdzJfi∗(R,z,ω)⋅Efi(R,z,ω)} (35)

which is Eq. 5. In the final line, the longitudinal momentum transfer appearing in **J***_fi_*(**R**, *z*, ω) and **E***_fi_*(**R**, *z*, ω) is locked to *q*_||_ = ω/*v_i_*. The explicit connection between this fully retarded form of the loss function and the quasistatic version in (*30*) is presented in the Supplementary Materials.

When the beam waist of the electron probe *w*_0_ is small compared to the length scale over which the response field changes, **E***_fi_* is approximately constant over the spatial domain where the current density is appreciable, allowing **E***_fi_*(**R**, *z*, ω) ≈ **E***_fi_*(**R**_0_, *z*, ω) in the last line of Eq. 35. The EEL probability in the narrow beam width limit, therefore, is

Γfi(R0,ω)≈−1πℏ2ωRe{∫dRdzJfi∗(R,z,ω)⋅Efi(R0,z,ω)}=−1πℏ2ωRe{∫dz[∫dRJfi∗(R,z,ω)]⋅Efi(R0,z,ω)}=−1πℏ2ωRe{∫dz[∫dRJfi∗(R,z,ω)eiωz/vi]e−iωz/vi⋅Efi(R0,z,ω)}=−1πℏ2ωRe{∫dz[∫dRJfi∗(R,ω)]e−iωz/vi⋅Efi(R0,z,ω)}=−1πℏ2ωRe{Jfi∗⋅∫dzEfi(R0,z,ω)e−iωz/vi} (36)

where $J_{fi}=\int dR\;J_{fi}(R,\omega )\;e^{-i\omega z/v_{i}}=(-e/mv_{i})\{\langle {\text{Ψ}}_{f}|{\hat{p}}_{⊥}|{\text{Ψ}}_{i}\rangle +[(2p_{i}-\hbar q_{∥})/2]\langle {\text{Ψ}}_{f}|{\text{Ψ}}_{i}\rangle \hat{z}\}$ is *z* independent. Additional details and analytic expressions for **J***_fi_*(**x**, ω) and $J_{fi}$ in the case of transitions between localized HG and LG transverse states are included in the Supplementary Materials.

## Supplementary Materials

### This PDF file includes:

Sections S1 to S6 Figs. S1 to S4
