# Supplementary material for: Optical polarization analogs in inelastic free-electron scattering
Source: Sci Adv. 2023 Dec 20;9(51):eadj6038. doi: 10.1126/sciadv.adj6038 (PMC10732523; doi:10.1126/sciadv.adj6038)
Supplement: Supplementary file 1 — Sections S1 to S6 Figs. S1 to S4 [file sciadv.adj6038_sm.pdf]

Science Advances
AAAS

Supplementary Materials for

Optical polarization analogs in inelastic free-electron scattering

Marc R. Bourgeois *et al.*

Corresponding author: David J. Masiello, masiello@uw.edu

Sci. Adv. **9**, eadj6038 (2023)
DOI: 10.1126/sciadv.adj6038

This PDF file includes:

Sections S1 to S6
Figs. S1 to S4

I. FULLY-RETARDED DOUBLE DIFFERENTIAL INELASTIC SCATTERING CROSS SECTION

A. Fully-Retarded, Single Dipole Case

This section presents the derivation of the fully-retarded double differential scattering cross section (DDSCS) for a single (in principle anisotropic) dipolar target. Starting from the equation for the DDSCS derived in the Materials and Methods,

$$\frac{\partial^2\sigma}{\partial E_{if}\partial\Omega} = -L^6\left(\frac{2m}{4\pi\hbar^2}\right)^2\int\frac{d(\hbar\omega)}{2\pi}\left(\frac{k_f}{k_i}\right)w_{fi}^{\text{loss}}(\omega), \tag{1}$$

where the frequency resolved electron energy loss rate is

$$\begin{aligned} w_{fi}^{\text{loss}}(\omega) &= \frac{4\pi^2}{\hbar}\int d\mathbf{x}\,d\mathbf{x}'\,\mathbf{J}_{fi}^*(\mathbf{x})\cdot\left[-\frac{2}{\pi}\text{Im}\left\{\overleftrightarrow{\mathbf{G}}(\mathbf{x},\mathbf{x}',\omega)\right\}\right]\cdot\mathbf{J}_{fi}(\mathbf{x}')\delta(\omega-\omega_{if}) \\ &= -\frac{8\pi}{\hbar}\int d\mathbf{x}\,d\mathbf{x}'\,\text{Im}\left\{\mathbf{J}_{fi}^*(\mathbf{x})\cdot\overleftrightarrow{\mathbf{G}}(\mathbf{x},\mathbf{x}',\omega)\cdot\mathbf{J}_{fi}(\mathbf{x}')\right\}\delta(\omega-\omega_{if}). \end{aligned} \tag{2}$$

For an isolated dipolar target with polarizability $\overleftrightarrow{\boldsymbol{\alpha}}(\omega)$ located at position $\mathbf{x}_d$, the induced Green's function can be expressed as (see Materials and Methods)

$$\overleftrightarrow{\mathbf{G}}(\mathbf{x},\mathbf{x}',\omega) = -\frac{1}{4\pi\omega^2}\overleftrightarrow{\tilde{\mathbf{G}}}_0(\mathbf{x},\mathbf{x}_d,\omega)\cdot\overleftrightarrow{\boldsymbol{\alpha}}(\omega)\cdot\overleftrightarrow{\tilde{\mathbf{G}}}_0(\mathbf{x}_d,\mathbf{x}',\omega), \tag{3}$$

where we have introduced

$$\overleftrightarrow{\tilde{\mathbf{G}}}_0(\mathbf{x},\mathbf{x}',\omega) = -4\pi\omega^2\overleftrightarrow{\mathbf{G}}_0(\mathbf{x},\mathbf{x}',\omega) = \left[\left(\frac{\omega}{c}\right)^2\overleftrightarrow{\mathbf{I}}+\nabla\nabla\right]\frac{e^{i\frac{\omega}{c}|\mathbf{x}-\mathbf{x}'|}}{|\mathbf{x}-\mathbf{x}'|}. \tag{4}$$

Beginning from the general expression for the DDSCS above,

$$\begin{aligned} \frac{\partial^2\sigma}{\partial E_{if}\partial\Omega} &= -L^6\left(\frac{2m}{4\pi\hbar^2}\right)^2\int\frac{d(\hbar\omega)}{2\pi}\left(\frac{k_f}{k_i}\right)w_{fi}^{\text{loss}}(\omega) \\ &= 4L^6\left(\frac{2m}{4\pi\hbar^2}\right)^2\iiint d\mathbf{x}\,d\mathbf{x}'\,d\omega\left(\frac{k_f}{k_i}\right)\text{Im}\left\{\mathbf{J}_{fi}^*(\mathbf{x})\cdot\overleftrightarrow{\mathbf{G}}(\mathbf{x},\mathbf{x}',\omega)\cdot\mathbf{J}_{fi}(\mathbf{x}')\right\}\delta(\omega-\omega_{if}) \\ &= -\frac{L^6}{\pi}\left(\frac{2m}{4\pi\hbar^2}\right)^2\iiint d\mathbf{x}\,d\mathbf{x}'\,d\omega\left(\frac{1}{\omega^2}\right)\left(\frac{k_f}{k_i}\right)\text{Im}\left\{\mathbf{J}_{fi}^*(\mathbf{x})\cdot\overleftrightarrow{\tilde{\mathbf{G}}}_0(\mathbf{x},\mathbf{x}_d,\omega)\cdot\overleftrightarrow{\boldsymbol{\alpha}}(\omega)\cdot\overleftrightarrow{\tilde{\mathbf{G}}}_0(\mathbf{x}_d,\mathbf{x}',\omega)\cdot\mathbf{J}_{fi}(\mathbf{x}')\right\}\delta(\omega-\omega_{if}) \\ &= -\frac{L^6m^2}{4\pi^3\hbar^4}\int d\omega\left(\frac{1}{\omega^2}\right)\left(\frac{k_f}{k_i}\right)\text{Im}\left\{\left[\int d\mathbf{x}\,\mathbf{J}_{fi}^*(\mathbf{x})\cdot\overleftrightarrow{\tilde{\mathbf{G}}}_0(\mathbf{x},\mathbf{x}_d,\omega)\right]\cdot\overleftrightarrow{\boldsymbol{\alpha}}(\omega)\cdot\left[\int d\mathbf{x}'\,\overleftrightarrow{\tilde{\mathbf{G}}}_0(\mathbf{x}_d,\mathbf{x}',\omega)\cdot\mathbf{J}_{fi}(\mathbf{x}')\right]\right\}\delta(\omega-\omega_{if}) \end{aligned} \tag{5}$$

To proceed, we specify to the case of incoming and outgoing plane wave electron states yielding a transition current density of the form $\mathbf{J}_{fi}(\mathbf{x}) = (-\hbar e/2mL^3)(2\mathbf{k}_i-\mathbf{q})e^{i\mathbf{q}\cdot\mathbf{x}}$. The spatial integrals involving the Green's Dyadic and transition current density can be evaluated using the Fourier transforms of the scalar Green's function $\int d\mathbf{x}'\,\frac{e^{+i\frac{\omega}{c}|\mathbf{x}'-\mathbf{x}|}}{|\mathbf{x}'-\mathbf{x}|}e^{\mp i\mathbf{q}\cdot\mathbf{x}'} =$

$-\frac{4\pi}{\frac{\omega^2}{c^2}-q^2}e^{\mp i\mathbf{q}\cdot\mathbf{x}}$. Explicitly,

$$\begin{aligned}\int d\mathbf{x}\,\mathbf{J}^*_{fi}(\mathbf{x})\cdot\overset{\leftrightarrow}{\widetilde{\mathbf{G}}}_0(\mathbf{x},\mathbf{x}_d,\omega) &= -\frac{\hbar e}{2mL^3}\int d\mathbf{x}\,(2\mathbf{k}^*_i-\mathbf{q}^*)e^{-i\mathbf{q}^*\cdot\mathbf{x}}\cdot\left[\left(\frac{\omega}{c}\right)^2\overset{\leftrightarrow}{\mathbf{I}}+\nabla_d\nabla_d\right]\frac{e^{i\frac{\omega}{c}|\mathbf{x}-\mathbf{x}_d|}}{|\mathbf{x}-\mathbf{x}_d|}\\ &= -\frac{\hbar e}{2mL^3}(2\mathbf{k}^*_i-\mathbf{q}^*)\cdot\left[\left(\frac{\omega}{c}\right)^2\overset{\leftrightarrow}{\mathbf{I}}+\nabla_d\nabla_d\right]\int d\mathbf{x}\,\frac{e^{i\frac{\omega}{c}|\mathbf{x}-\mathbf{x}_d|}}{|\mathbf{x}-\mathbf{x}_d|}e^{-i\mathbf{q}^*\cdot\mathbf{x}}\\ &= \frac{2\pi\hbar e}{mL^3}(2\mathbf{k}^*_i-\mathbf{q}^*)\cdot\left[\left(\frac{\omega}{c}\right)^2\overset{\leftrightarrow}{\mathbf{I}}-\mathbf{q}^*\mathbf{q}^*\right]\frac{e^{-i\mathbf{q}^*\cdot\mathbf{x}_d}}{\frac{\omega^2}{c^2}-q^2},\end{aligned}\tag{6}$$

and, similarly,

$$\int d\mathbf{x}'\,\overset{\leftrightarrow}{\widetilde{\mathbf{G}}}_0(\mathbf{x}_d,\mathbf{x}',\omega)\cdot\mathbf{J}_{fi}(\mathbf{x}') = -\frac{2\pi\hbar e}{mL^3}(2\mathbf{k}_i-\mathbf{q})\cdot\left[\left(\frac{\omega}{c}\right)^2\overset{\leftrightarrow}{\mathbf{I}}-\mathbf{q}\mathbf{q}\right]\frac{e^{i\mathbf{q}\cdot\mathbf{x}_d}}{\frac{\omega^2}{c^2}-q^2}.\tag{7}$$

Therefore,

$$\begin{aligned}\frac{\partial^2\sigma}{\partial E_{if}\partial\Omega} &= \frac{e^2k_f}{\hbar^2\pi k_i}\int\frac{d\omega}{\omega^2}\,\mathrm{Im}\left\{\left[\int d\mathbf{x}\,\mathbf{J}^*_{fi}(\mathbf{x})\cdot\overset{\leftrightarrow}{\widetilde{\mathbf{G}}}_0(\mathbf{x},\mathbf{x}_d,\omega)\right]\cdot\overset{\leftrightarrow}{\boldsymbol{\alpha}}(\omega)\cdot\left[\int d\mathbf{x}'\,\overset{\leftrightarrow}{\widetilde{\mathbf{G}}}_0(\mathbf{x}_d,\mathbf{x}',\omega)\cdot\mathbf{J}_{fi}(\mathbf{x}')\right]\right\}\delta(\omega-\omega_{if})\\ &= \frac{e^2k_f}{\hbar^2\pi k_i}\int\frac{d\omega}{\omega^2}\,\mathrm{Im}\left\{\mathbf{Q}^*\cdot\left[\left(\frac{\omega}{c}\right)^2\overset{\leftrightarrow}{\mathbf{I}}-\mathbf{q}^*\mathbf{q}^*\right]\frac{e^{-i\mathbf{q}^*\cdot\mathbf{x}_d}}{\left(\frac{\omega^2}{c^2}-q^2\right)^*}\cdot\overset{\leftrightarrow}{\boldsymbol{\alpha}}(\omega)\cdot\left[\left(\frac{\omega}{c}\right)^2\overset{\leftrightarrow}{\mathbf{I}}-\mathbf{q}\mathbf{q}\right]\frac{e^{i\mathbf{q}\cdot\mathbf{x}_d}}{\frac{\omega^2}{c^2}-q^2}\cdot\mathbf{Q}\right\}\delta(\omega-\omega_{if})\\ &= \frac{e^2k_f}{\hbar^2\pi k_i}\int\frac{d\omega}{\omega^2}\,\mathrm{Im}\left\{\mathbf{Q}^*\cdot\left[\left(\frac{\omega}{c}\right)^2\overset{\leftrightarrow}{\mathbf{I}}-\mathbf{q}^*\mathbf{q}^*\right]\cdot\frac{\overset{\leftrightarrow}{\boldsymbol{\alpha}}(\omega)\,e^{i(\mathbf{q}-\mathbf{q}^*)\cdot\mathbf{x}_d}}{\left|\frac{\omega^2_{if}}{c^2}-q^2\right|^2}\cdot\left[\left(\frac{\omega}{c}\right)^2\overset{\leftrightarrow}{\mathbf{I}}-\mathbf{q}\mathbf{q}\right]\cdot\mathbf{Q}\right\}\delta(\omega-\omega_{if})\\ &= \frac{e^2k_f}{\hbar^2\pi k_i}\frac{1}{\omega^2_{if}}\,\mathrm{Im}\left\{\mathbf{Q}^*\cdot\left[\left(\frac{\omega_{if}}{c}\right)^2\overset{\leftrightarrow}{\mathbf{I}}-\mathbf{q}^*\mathbf{q}^*\right]\cdot\frac{\overset{\leftrightarrow}{\boldsymbol{\alpha}}(\omega_{if})\,e^{i(\mathbf{q}-\mathbf{q}^*)\cdot\mathbf{x}_d}}{\left|\frac{\omega^2_{if}}{c^2}-q^2\right|^2}\cdot\left[\left(\frac{\omega_{if}}{c}\right)^2\overset{\leftrightarrow}{\mathbf{I}}-\mathbf{q}\mathbf{q}\right]\cdot\mathbf{Q}\right\}\end{aligned}\tag{8}$$

where $\mathbf{Q}=2\mathbf{k}_i-\mathbf{q}$.

B. Quasistatic Limit

In the quasistatic ($c\to\infty$) limit with the dipolar target located at the origin ($\mathbf{x}_d=\mathbf{0}$), Eq. (8) reduces to

$$\begin{aligned}\frac{\partial^2\sigma}{\partial E_{if}\partial\Omega} &= \frac{e^2k_f}{\hbar^2\pi k_i}\frac{1}{\omega^2_{if}}\,\mathrm{Im}\left\{\mathbf{Q}^*\cdot\left[\left(\frac{\omega_{if}}{c}\right)^2\overset{\leftrightarrow}{\mathbf{I}}-\mathbf{q}^*\mathbf{q}^*\right]\cdot\frac{\overset{\leftrightarrow}{\boldsymbol{\alpha}}(\omega_{if})}{\left|\frac{\omega^2_{if}}{c^2}-q^2\right|^2}\cdot\left[\left(\frac{\omega_{if}}{c}\right)^2\overset{\leftrightarrow}{\mathbf{I}}-\mathbf{q}\mathbf{q}\right]\cdot\mathbf{Q}\right\}\\ &= \frac{e^2k_f}{\hbar^2\pi k_i}\frac{1}{\omega^2_{if}}\,\mathrm{Im}\left\{\mathbf{Q}^*\cdot\mathbf{q}^*\mathbf{q}^*\cdot\frac{\overset{\leftrightarrow}{\boldsymbol{\alpha}}(\omega_{if})}{q^4}\cdot\mathbf{q}\mathbf{q}\cdot\mathbf{Q}\right\}.\end{aligned}\tag{9}$$

By leveraging the non-relativistic kinematic relationship $\mathbf{Q}\cdot\mathbf{q}=2(\mathbf{k}_i\cdot\mathbf{q})-\mathbf{q}\cdot\mathbf{q}=\mathbf{k}^2_i-\mathbf{k}^2_f=(2m/\hbar)\omega_{if}$ this becomes

$$\frac{\partial^2\sigma}{\partial E_{if}\partial\Omega}=\frac{4m^2e^2}{q^4\hbar^4\pi}\left(\frac{k_f}{k_i}\right)\mathrm{Im}\left\{\mathbf{q}^*\cdot\overset{\leftrightarrow}{\boldsymbol{\alpha}}(\omega_{if})\cdot\mathbf{q}\right\}.\tag{10}$$

We emphasize that this expression is derived by taking a macroscopic QED perspective of the target, with fully-retarded electromagnetic coupling between target and probing free electron. This is in contrast to the more conventional approach, where a microscopic perspective is taken with j bound target electrons interacting with the probing

free electron via the Coulomb interaction. The microscopic approach, in the small target ($\mathbf{q}\cdot\mathbf{x}_j \ll 1$) limit, leads to the "dipole approximation" for EELS [6,8], where the double differential cross section for electron scattering from a target with a single bound electron is

$$\frac{\partial^2\sigma}{\partial E_{if}\partial\Omega} = \sum_b \frac{4}{a_0^2}\left(\frac{k_f}{k_i}\right)\frac{1}{q^4}\big|\left\langle b\right| i\mathbf{q}\cdot\hat{\mathbf{x}}\left|a\right\rangle\big|^2\delta(E_b - E_a - E_{if}). \tag{11}$$

Here, a is the target ground state, b labels the target excited states. Noting that $\big|\left\langle b\right| i\mathbf{q}\cdot\hat{\mathbf{x}}\left|a\right\rangle\big|^2 = \big(\left\langle b\right| i\mathbf{q}\cdot\hat{\mathbf{x}}\left|a\right\rangle\big)^*\left\langle b\right| i\mathbf{q}\cdot\hat{\mathbf{x}}\left|a\right\rangle = -i\mathbf{q}^*\cdot\left\langle a\right|\hat{\mathbf{x}}\left|b\right\rangle\left\langle b\right|\hat{\mathbf{x}}\left|a\right\rangle\cdot i\mathbf{q} = q_l^*\left\langle a\right|\hat{x}_l\left|b\right\rangle\left\langle b\right|\hat{x}_j\left|a\right\rangle q_j$, and using the relationship $\frac{1}{\omega\pm i\eta} = \mathcal{P}\left(\frac{1}{\omega}\right)\mp i\pi\delta(\omega)$, it is possible to write

$$\begin{aligned}\sum_b \big|\left\langle b\right| i\mathbf{q}\cdot\hat{\mathbf{x}}\left|a\right\rangle\big|^2\delta(E_{if} - E_{ba}) &= \frac{1}{\pi e^2}\ q_l^*\ \mathrm{Im}\left\{\sum_b\frac{\left\langle a\right|\hat{\mu}_l\left|b\right\rangle\left\langle b\right|\hat{\mu}_j\left|a\right\rangle}{E_{if}-E_{ba}}\right\}\ q_j\\ &= \frac{1}{\pi e^2}\ q_l^*\ \mathrm{Im}\left\{\alpha_{lj}^{aa}(\omega_{if})\right\}\ q_j\\ &= \frac{1}{\pi e^2}\ \mathbf{q}^*\cdot\mathrm{Im}\left\{\overleftrightarrow{\boldsymbol{\alpha}}^{aa}(E_{if})\right\}\cdot\mathbf{q},\end{aligned} \tag{12}$$

where $\hat{\mu}_l = e\hat{x}_l$ is the lth Cartesian component of the dipole operator and $\overleftrightarrow{\boldsymbol{\alpha}}^{aa}(E_{if})$ is the ground state Kramers-Heisenberg polarizability tensor evaluated at the loss energy E_{if}. Putting this all together, we find the double differential inelastic scattering cross section for a single bound target electron within the quasistatic dipole approximation to be

$$\frac{\partial^2\sigma^{\mathrm{dip}}}{\partial E_{if}\partial\Omega} = \frac{4m^2e^2}{\pi\hbar^4}\left(\frac{k_f}{k_i}\right)\frac{1}{q^4}\ \mathbf{q}^*\cdot\mathrm{Im}\left\{\overleftrightarrow{\boldsymbol{\alpha}}^{aa}(E_{if})\right\}\cdot\mathbf{q}, \tag{13}$$

which is in agreement with the quasistatic $c\to\infty$ limit form of $\partial^2\sigma/\partial E_{if}\partial\Omega$ given by Eq. (10).

II. STATE- AND ENERGY-RESOLVED EEL PROBABILITY $\Gamma_{fi}(\mathbf{R},\omega)$

A. Recovery of Quasistatic Loss Function

This section outlines the recovery of the energy-resolved loss probability given in Ref. [30], where the electron-target interaction is treated quasistatically. In the $c\to\infty$ limit, the Green's dyadic is related to the screened interaction $W(\mathbf{x},\mathbf{x}',\omega)$ by $4\pi\omega^2\overleftrightarrow{\mathbf{G}}(\mathbf{x},\mathbf{x}',\omega) = \nabla\nabla' W(\mathbf{x},\mathbf{x}',\omega)$ [30]. As discussed in the Materials and Methods section, the loss function can be written as

$$\begin{aligned}\Gamma_{fi}(\mathbf{R},\omega) &= -\frac{4L^2}{\hbar^2v^2}\mathrm{Im}\left\{\int d\mathbf{x}\,d\mathbf{x}'\,\mathbf{J}_{fi}^*(\mathbf{x})\cdot\overleftrightarrow{\mathbf{G}}(\mathbf{x},\mathbf{x}',\omega)\cdot\mathbf{J}_{fi}(\mathbf{x}')\right\},\\ &= -\frac{4L^2}{\hbar^2v^2}\mathrm{Im}\left\{\int d\mathbf{x}\,d\mathbf{x}'\,\mathbf{J}_{fi}^*(\mathbf{x})\cdot\left[\frac{1}{4\pi\omega^2}\nabla\nabla' W(\mathbf{x},\mathbf{x}',\omega)\right]\cdot\mathbf{J}_{fi}(\mathbf{x}')\right\},\end{aligned} \tag{14}$$

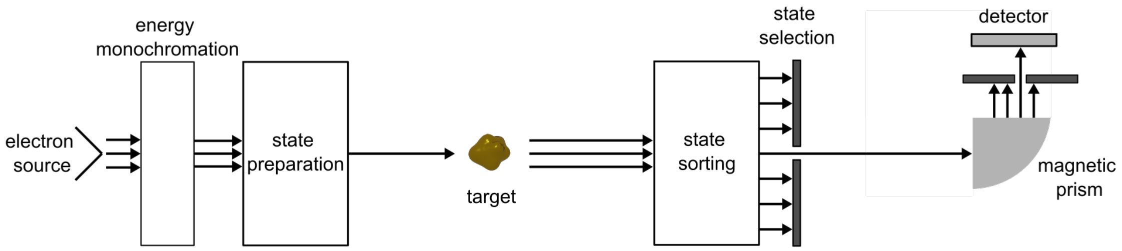

FIG. S1. **Experimental scheme depicting the sequential ordering of essential elements involved in a phase-shaped EELS measurement.** See Section III for additional discussion.

which can be integrated twice by parts to obtain

$$\Gamma_{fi}(\mathbf{R},\omega) = -\frac{4L^2}{\hbar^2 v^2}\frac{1}{4\pi\omega^2}\mathrm{Im}\left\{\int d\mathbf{x}\,d\mathbf{x}'\,\left[\nabla\cdot\mathbf{J}^*_{fi}(\mathbf{x},\omega)\right]W(\mathbf{x},\mathbf{x}',\omega)\left[\nabla'\cdot\mathbf{J}_{fi}(\mathbf{x}',\omega)\right]\right\}. \tag{15}$$

In the case of harmonic time dependence, the continuity equation $\dot{\rho}_{fi}(\mathbf{x},t) = -\nabla\cdot\mathbf{J}_{fi}(\mathbf{x},t)$ becomes $-i\omega_{if}\rho_{fi}(\mathbf{x}) = -\nabla\cdot\mathbf{J}_{fi}(\mathbf{x})$, with $\rho_{fi}(\mathbf{x}) = -e\psi^*_f(\mathbf{x})\psi_i(\mathbf{x}) = (-e/L)\Psi^*_f(\mathbf{x})\Psi_i(\mathbf{x})e^{iq_\parallel z}$. This allows the loss function to be rewritten as

$$\Gamma_{fi}(\mathbf{R},\omega) = \frac{e^2}{\pi\hbar^2 v^2}\int d\mathbf{x}\,d\mathbf{x}'\,\Psi_f(\mathbf{x})\Psi^*_i(\mathbf{x})e^{-iq_\parallel z}\mathrm{Im}\left\{-W(\mathbf{x},\mathbf{x}',\omega)\right\}\Psi^*_f(\mathbf{x}')\Psi_i(\mathbf{x}')e^{iq_\parallel z'}, \tag{16}$$

which is equivalent to Eq. (1) of Ref. [30].

B. Equivalence between $\hat{\boldsymbol{\mathcal{J}}}^\perp_{fi}$ and $\hat{\mathbf{d}}^\perp_{fi}$ Optical Polarization Analogs

Ref. [30] identifies $\hat{\mathbf{d}}^\perp_{fi}$ as an OPA in inelastic free electron scattering processes, where $\mathbf{d}^\perp_{fi} = -\langle\Psi_f|e\mathbf{x}_\perp|\Psi_i\rangle$. Within the small w_0 narrow beam limit,

$$\begin{aligned}\boldsymbol{\mathcal{J}}^\perp_{fi} &= \int d\mathbf{R}\mathbf{J}^\perp_{fi}(\mathbf{R},z)e^{-i\omega z/v}\\ &= (i\hbar e/2mv_i)\left[\langle\Psi_f|\nabla_\perp|\Psi_i\rangle - \langle\Psi_i|\nabla_\perp|\Psi_f\rangle^*\right]\\ &= (i\hbar e/mv_i)\langle\Psi_f|\nabla_\perp|\Psi_i\rangle\,.\end{aligned} \tag{17}$$

Suppose the initial transverse state is the Gaussian state $\Psi^{\mathrm{HG}}_{00}(x,y)$ and the final state resides on the surface of the Poincaré sphere shown Fig. 2F in the main text. Using the fact that $(\partial/\partial x)\Psi^{\mathrm{HG}}_{00}(x,y) = (-1/w_0)\Psi^{\mathrm{HG}}_{10}(x,y)$ and $(\partial/\partial y)\Psi^{\mathrm{HG}}_{00}(x,y) = (-1/w_0)\Psi^{\mathrm{HG}}_{01}(x,y)$, one finds $\hat{\boldsymbol{\mathcal{J}}}^\perp_{fi}\parallel\hat{\mathbf{d}}^\perp_{fi}$.

III. EXPERIMENTAL MEASUREMENT SCHEMES

Figure S1 presents a scheme showing the sequential ordering of essential elements required to perform a phase-shaped EELS measurement. Figure 2A and 2B of the main text presented one possible measurement scheme for delocalized and localized transverse states, respectively. As discussed in the main text and elsewhere within the Materials and Methods, if the initial and final free electron wave functions separate into longitudinal and transverse

parts with the form $\psi(\mathbf{x}) = \Psi(\mathbf{x}_\perp) L^{-1/2} e^{ik_3 x_3}$, then the transition current density also separates into longitudinal and transverse parts according to $\mathbf{J}_{fi}(\mathbf{x}) = \big[\mathbf{J}^\perp_{fi}(\mathbf{x}_\perp) + J^\parallel_{fi}(\mathbf{x}_\perp)\hat{\mathbf{x}}_3\big] e^{iq_\parallel x_3}$, where $q_\parallel = k^i_3 - k^f_3$ is the longitudinal momentum transfer, and

$$\begin{aligned}\mathbf{J}^\perp_{fi}(\mathbf{x}_\perp) &= \frac{i\hbar e}{2mL}\Big\{\Psi^*_f(\mathbf{x}_\perp)\nabla_\perp\Psi_i(\mathbf{x}_\perp) - \Psi_i(\mathbf{x}_\perp)\nabla_\perp\Psi^*_f(\mathbf{x}_\perp)\Big\}\\ J^\parallel_{fi}(\mathbf{x}_\perp) &= -\frac{\hbar e}{2mL}(2k^i_3 - q_\parallel)\Psi^*_f(\mathbf{x}_\perp)\Psi_i(\mathbf{x}_\perp).\end{aligned} \tag{18}$$

It it evident from this definition that interchanging the initial and final transverse states while retaining the identities of the longitudinal components of the initial and final states, then $\mathbf{J}^\perp_{fi}(\mathbf{x}_\perp) \to \mathbf{J}^\perp_{if}(\mathbf{x}_\perp) = \mathbf{J}^{\perp *}_{fi}(\mathbf{x}_\perp)$.

The EEL rate w_{fi} is proportional to $\int d\mathbf{x}\, d\mathbf{x}' \mathbf{J}^*_{fi}(\mathbf{x}) \cdot \overleftrightarrow{\mathbf{G}}(\mathbf{x}, \mathbf{x}', \omega) \cdot \mathbf{J}_{fi}(\mathbf{x}')$. By interchanging the 3D coordinates $\mathbf{x}$ and $\mathbf{x}'$, and invoking reciprocity, i.e., $G_{\alpha\beta}(\mathbf{x}, \mathbf{x}', \omega) = G_{\beta\alpha}(\mathbf{x}', \mathbf{x}, \omega)$,

$$\begin{aligned}\int d\mathbf{x}\, d\mathbf{x}' \big[\mathbf{J}_{fi}(\mathbf{R}) e^{iq_\parallel z}\big]^*_\alpha G_{\alpha\beta}(\mathbf{x}, \mathbf{x}', \omega)\big[\mathbf{J}_{fi}(\mathbf{R}') e^{iq_\parallel z'}\big]_\beta &= \int d\mathbf{x}\, d\mathbf{x}' \big[\mathbf{J}_{fi}(\mathbf{R})\big]^*_\alpha G_{\alpha\beta}(\mathbf{x}, \mathbf{x}', \omega)\big[\mathbf{J}_{fi}(\mathbf{R}')\big]_\beta e^{-iq_\parallel(z-z')}\\ &= \int d\mathbf{x}\, d\mathbf{x}' \big[\mathbf{J}_{if}(\mathbf{R})\big]_\alpha G_{\alpha\beta}(\mathbf{x}, \mathbf{x}', \omega)\big[\mathbf{J}_{if}(\mathbf{R}')\big]^*_\beta e^{-iq_\parallel(z-z')}\\ &= \int d\mathbf{x}\, d\mathbf{x}' \big[\mathbf{J}_{if}(\mathbf{R}')\big]^*_\beta G_{\beta\alpha}(\mathbf{x}', \mathbf{x}, \omega)\big[\mathbf{J}_{if}(\mathbf{R})\big]_\alpha e^{-iq_\parallel(z-z')}\\ &= \int d\mathbf{x}\, d\mathbf{x}' \big[\mathbf{J}_{if}(\mathbf{R})\big]^*_\alpha G_{\alpha\beta}(\mathbf{x}, \mathbf{x}', \omega)\big[\mathbf{J}_{if}(\mathbf{R}')\big]_\beta e^{-iq_\parallel(z'-z)}\\ &= \int d\mathbf{x}\, d\mathbf{x}' \big[\mathbf{J}_{if}(\mathbf{R}) e^{i(-q_\parallel)z}\big]^*_\alpha G_{\alpha\beta}(\mathbf{x}, \mathbf{x}', \omega)\big[\mathbf{J}_{if}(\mathbf{R}') e^{i(-q_\parallel)z'}\big]_\beta,\end{aligned} \tag{19}$$

where $\alpha, \beta = x, y, z$ and Einstein summation notation has been used. Eq. (19) can be equivalently expressed as $w_{fi}(q_\parallel) = w_{if}(-q_\parallel)$ provided $G_{\alpha\beta}(\mathbf{x}, \mathbf{x}', \omega) = G_{\beta\alpha}(\mathbf{x}', \mathbf{x}, \omega)$. Said differently, interchanging the initial and final transverse states together with changing the sign of the recoil momentum wave vector $q_\parallel$ leaves the EEL observable invariant in a reciprocal medium. In the case of an isolated dipolar target at position $\mathbf{x}_d$ characterized by frequency-dependent polarizability $\overleftrightarrow{\boldsymbol{\alpha}}(\omega)$, $\overleftrightarrow{\mathbf{G}}(\mathbf{x}, \mathbf{x}', \omega)$ satisfies the reciprocity condition when the polarizability tensor is complex symmetric, i.e., when $\overleftrightarrow{\boldsymbol{\alpha}}(\omega) = \overleftrightarrow{\boldsymbol{\alpha}}^T(\omega)$. Specifically, the $\alpha\beta$-component of $\overleftrightarrow{\mathbf{G}}(\mathbf{x}, \mathbf{x}', \omega)$ is

$$\begin{aligned}G_{\alpha\beta}(\mathbf{x}, \mathbf{x}', \omega) &= -4\pi\omega^2 \widetilde{G}^0_{\alpha\gamma}(\mathbf{x}, \mathbf{x}_d, \omega)\alpha_{\gamma\gamma'}(\omega)\widetilde{G}^0_{\gamma'\beta}(\mathbf{x}_d, \mathbf{x}', \omega)\\ &= -4\pi\omega^2 \widetilde{G}^0_{\beta\gamma'}(\mathbf{x}', \mathbf{x}_d, \omega)\alpha_{\gamma\gamma'}(\omega)\widetilde{G}^0_{\gamma\alpha}(\mathbf{x}_d, \mathbf{x}, \omega).\end{aligned} \tag{20}$$

Evidently, $\overleftrightarrow{\mathbf{G}}(\mathbf{x}, \mathbf{x}', \omega) = \overleftrightarrow{\mathbf{G}}^T(\mathbf{x}', \mathbf{x}, \omega)$ is guaranteed provided that the polarizability tensor is a (complex) symmetric matrix.

IV. NUMERICAL CALCULATIONS

As described in the main text and derived in the Materials and Methods section, both $\partial^2\sigma/\partial E_{if}\partial\Omega$ and Γ_{fi} observables are dictated by the state- and frequency-resolved loss rate

$$w_{fi}^{\rm loss}(\omega) = \frac{-8\pi}{\hbar}{\rm Im}\left\{\int d\mathbf{x}\, d\mathbf{x}'\, \mathbf{J}^*_{fi}(\mathbf{x})\cdot \overleftrightarrow{\mathbf{G}}(\mathbf{x},\mathbf{x}',\omega)\cdot \mathbf{J}_{fi}(\mathbf{x}')\right\}\delta(\omega-\omega_{if}). \tag{21}$$

Using the coupled-dipole propagator identity

$$\overleftrightarrow{\mathbf{G}}(\mathbf{x},\mathbf{x}',\omega) = \sum_{jj'}(+4\pi i\omega)\overleftrightarrow{\mathbf{G}}_0(\mathbf{x},\mathbf{x}_j,\omega)\cdot\left(\overleftrightarrow{\boldsymbol{\alpha}}^{-1}+4\pi\omega^2\overleftrightarrow{\mathbf{G}}_0\right)^{-1}_{jj'}\cdot(-4\pi i\omega)\overleftrightarrow{\mathbf{G}}_0(\mathbf{x}'_{j'},\mathbf{x}',\omega), \tag{22}$$

which is introduced in the Materials and Methods section, the portion of Eq. (21) inside the imaginary operation can be expressed as

$$\begin{aligned}
&\int d\mathbf{x}\, d\mathbf{x}'\, \mathbf{J}^*_{fi}(\mathbf{x})\cdot \overleftrightarrow{\mathbf{G}}(\mathbf{x},\mathbf{x}',\omega)\cdot \mathbf{J}_{fi}(\mathbf{x}') = \\
&= 16\pi^2\omega^2\sum_{jj'}\int d\mathbf{x}\, d\mathbf{x}'\, \mathbf{J}^*_{fi}(\mathbf{x})\cdot\left[\overleftrightarrow{\mathbf{G}}_0(\mathbf{x},\mathbf{x}_j,\omega)\cdot\left(\overleftrightarrow{\boldsymbol{\alpha}}^{-1}+4\pi\omega^2\overleftrightarrow{\mathbf{G}}_0\right)^{-1}_{jj'}\cdot\overleftrightarrow{\mathbf{G}}_0(\mathbf{x}'_{j'},\mathbf{x}',\omega)\right]\cdot\mathbf{J}_{fi}(\mathbf{x}') \\
&= 16\pi^2\omega^2\sum_{j}\int d\mathbf{x}\, \mathbf{J}^*_{fi}(\mathbf{x})\cdot\overleftrightarrow{\mathbf{G}}_0(\mathbf{x},\mathbf{x}_j,\omega)\cdot\sum_{j'}\left(\overleftrightarrow{\boldsymbol{\alpha}}^{-1}+4\pi\omega^2\overleftrightarrow{\mathbf{G}}_0\right)^{-1}_{jj'}\cdot\int d\mathbf{x}'\,\overleftrightarrow{\mathbf{G}}_0(\mathbf{x}'_{j'},\mathbf{x}',\omega)\cdot\mathbf{J}_{fi}(\mathbf{x}') \\
&= 16\pi^2\omega^2\sum_{j}\left(\frac{v}{4Li\pi\omega}\right)\mathbf{E}^{0*}_{fi}(\mathbf{x}_j,\omega)\cdot\sum_{j'}\left(\overleftrightarrow{\boldsymbol{\alpha}}^{-1}+4\pi\omega^2\overleftrightarrow{\mathbf{G}}_0\right)^{-1}_{jj'}\cdot\left(-\frac{v}{4Li\pi\omega}\right)\mathbf{E}^{0}_{fi}(\mathbf{x}'_{j'},\omega) \\
&= \frac{v^2}{L^2}\sum_{j}\mathbf{E}^{0*}_{fi}(\mathbf{x}_j,\omega)\cdot\sum_{j'}\left(\overleftrightarrow{\boldsymbol{\alpha}}^{-1}+4\pi\omega^2\overleftrightarrow{\mathbf{G}}_0\right)^{-1}_{jj'}\cdot\,\mathbf{E}^{0}_{fi}(\mathbf{x}'_{j'},\omega) \\
&= \frac{v^2}{L^2}\sum_{j}\mathbf{E}^{0*}_{fi}(\mathbf{x}_j,\omega)\cdot\mathbf{p}^j_{fi}
\end{aligned} \tag{23}$$

where $\mathbf{E}^0_{fi}(\mathbf{x},\omega) = -(4L\pi i\omega/v)\int d\mathbf{x}'\,\overleftrightarrow{\mathbf{G}}_0(\mathbf{x},\mathbf{x}',\omega)\cdot\mathbf{J}_{fi}(\mathbf{x}')$ and the induced dipole moments $\mathbf{p}^j_{fi}$ are

$$\mathbf{p}^j_{fi} = \sum_{j'}\left(\overleftrightarrow{\boldsymbol{\alpha}}^{-1}+4\pi\omega^2\overleftrightarrow{\mathbf{G}}_0\right)^{-1}_{jj'}\cdot\,\mathbf{E}^0_{fi}(\mathbf{x}'_j,\omega). \tag{24}$$

We modified the e-DDA discrete dipole code [42, 43] to accommodate the transition fields $\mathbf{E}^0_{fi}$ described in Sections V and V B relevant to the transverse state transitions depicted in Fig. 2 of the main text. Once the induced dipole moments are determined using e-DDA, the $\partial^2\sigma/\partial E_{if}\partial\Omega$ and Γ_{fi} observables are evaluated using Eq. 4 in the main text.

In the case of plane wave initial and final free electron states, the non-recoil approximation is not invoked as it was when dealing with transversely localized states as discussed in the Materials and Methods section. Instead, relativistic kinematics is employed as described briefly here. The total relativistic energy (kinetic energy + rest mass energy) of the incoming free electron is $E_i = \gamma_i mc^2$, where $\gamma_i = (1-\beta_i^2)^{-1/2}$ is the Lorentz factor, and $\beta = v/c$. The form of the final electron energy is identical with $i\to f$, and $E_{if} = E_i - E_f = (\gamma_i-\gamma_f)mc^2$. Knowing the loss energy E_{if} and the

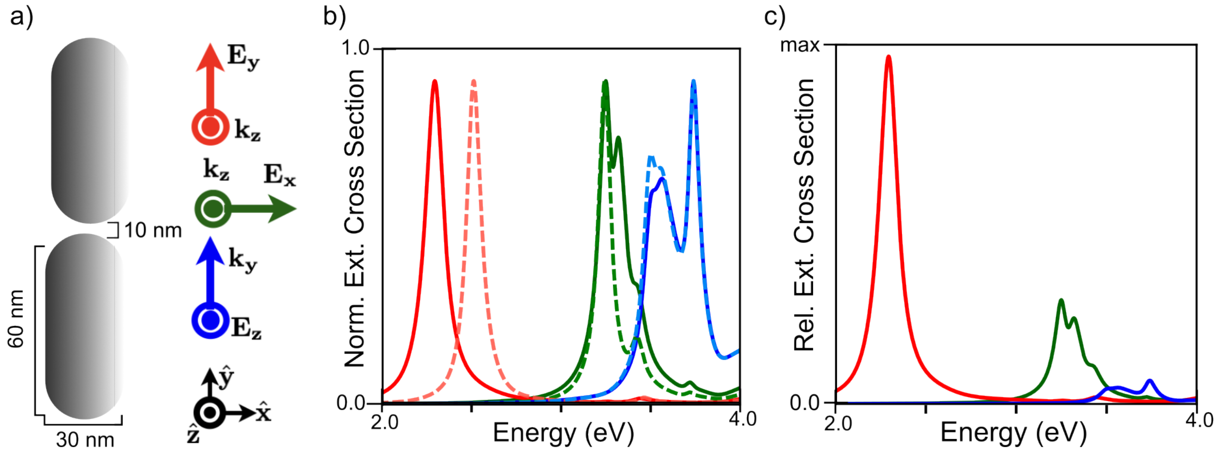

FIG. S2. **Schematic and optical extinction spectra of Ag rod dimer system considered in Fig. 3 of the main text.** (**A**) Schematic of the 30 nm × 60 nm × 15 nm rod dimer with color-coded diagrams indicating the excitation conditions for panels (B) and (c). (**B**) Normalized optical extinction cross section spectra for the rod dimer (solid) and single rod (dashed) systems. (**C**) Relative optical extinction cross section spectra for the rod dimer in (B).

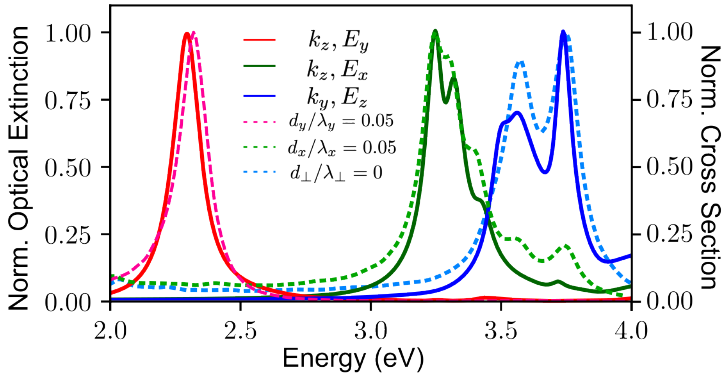

FIG. S3. **Optical extinction and inelastic electron scattering double differential cross section spectra.** Solid lines are normalized optical extinction spectra reproduced from Fig. S2. Dashed traces are normalized $\partial^2\sigma/\partial E_{if}\partial\Omega$ extracted from main text Fig. 3A,B within the dipole limit at $d/\lambda_\perp = 0.05$.

incoming electron speed, the final electron speed can be determined using $\gamma_f = \gamma_i - (E_{if}/mc^2)$ and $\beta_f = \sqrt{1-\gamma_f^{-2}}$. This fixes the magnitude of the outgoing wave vector $k_f = m\gamma_f\beta_f c/\hbar$. The observation angle θ is then selected such that $\mathbf{k}_f = k_f \cos\theta\ \hat{\mathbf{z}} + k_f \sin\theta\ \hat{\mathbf{x}}_\perp$, and $\mathbf{q} = (k_i - k_f\cos\theta)\hat{\mathbf{z}} - k_f \sin\theta\ \hat{\mathbf{x}}_\perp$.

Fig. 3 in the main text presents numerically calculated phase-shaped EEL observables for an Ag rod dimer system comprised of two 30 nm × 60 nm × 15 nm rods with a 10 nm surface-to-surface gap along the $\hat{\mathbf{y}}$ axis (Fig. S2A). Fig. S2B shows normalized optical extinction cross section spectra $\sigma_{\text{ext}}(\omega)$ calculated using DDSCAT [47] for the rod dimer (solid) and individual rod (dashed) systems excited by plane wave light polarized along $\hat{\mathbf{x}}$ (green), $\hat{\mathbf{y}}$ (red), and $\hat{\mathbf{z}}$ (blue). The amplitudes of the dimer cross section spectra in panel (b) relative to the largest (red) are shown in Fig. S2C. Fig. S3 shows $\partial^2\sigma/\partial E_{if}\partial\Omega$ for the rod dimer system considered in Fig. S2 for $\mathbf{q}_\perp = \mathbf{0}$ (dashed blue) as well as for $\mathbf{q}_\perp \neq \mathbf{0}$ and $\hat{\mathbf{q}}$ oriented along $\hat{\mathbf{x}}$ (red dashed) and $\hat{\mathbf{y}}$ (green dashed) at observation angles θ (see main text Fig. 3) corresponding to $d/\lambda_\perp = 0.05$. The normalized optical extinction cross section spectra $\sigma_{\text{ext}}(\omega)$ from Fig. S2B (solid) are included for comparison.

V. TRANSITION CURRENTS $\mathbf{J}_{fi}$ AND ASSOCIATED TRANSITION FIELDS $\mathbf{E}^0_{fi}$

A. Delocalized Plane Wave States

The transverse transition current density arising from transitioning from $|\mathbf{k}_\perp\rangle = |0\,0\rangle$ to an arbitrary superposition of states on the surface of the transition sphere in Fig. 2E is $\mathbf{J}^\perp_{fi}(\mathbf{x}) = (\hbar e/2mL^2)e^{iq_\parallel x_3}\int_{|\mathbf{k}_\perp|=q_\perp}\mathbf{k}_\perp d\mathbf{k}_\perp \widetilde{\Psi}^*_f(\mathbf{k}_\perp)e^{-i\mathbf{k}_\perp\cdot\mathbf{x}_\perp}/(2\pi)^2$, where $\widetilde{\Psi}_f(\mathbf{k}_\perp)$ is the reciprocal space wave function of the final 2D transverse pure state and L is the box quantization length. If the final state consists of a single plane wave component, $\mathbf{J}_{fi}(\mathbf{x}) = (-\hbar e/2mL^3)(2\mathbf{k}_i - \mathbf{q})e^{i\mathbf{q}\cdot\mathbf{x}}$.

The free space transition field, i.e., the electric field sourced by the transition current density in the absence of a target, is

$$\mathbf{E}^0_{fi}(\mathbf{x},\omega) = -4i\pi\omega\int d\mathbf{x}'\,\overleftrightarrow{\mathbf{G}}_0(\mathbf{x},\mathbf{x}',\omega)\cdot\left(\frac{L}{v}\right)\mathbf{J}_{fi}(\mathbf{x}'). \tag{25}$$

Using the Fourier transform of the scalar Green's function $\int d\mathbf{x}'\,\frac{e^{i\frac{\omega}{c}|\mathbf{x}'-\mathbf{x}|}}{|\mathbf{x}'-\mathbf{x}|}e^{\mp i\mathbf{q}\cdot\mathbf{x}'} = -\frac{4\pi}{\frac{\omega^2}{c^2}-q^2}e^{\mp i\mathbf{q}\cdot\mathbf{x}}$, we can perform the necessary spatial integration in Eq. (25) to obtain an analytical form for the induced electric field (with units statV·cm^{-1}·s),

$$\begin{aligned}
\mathbf{E}^0_{fi}(\mathbf{x},\omega) &= \frac{i}{\omega}\int d\mathbf{x}'\left\{\left(\frac{\omega}{c}\right)^2\overleftrightarrow{\mathbf{I}} + \nabla\nabla\right\}\frac{e^{i\frac{\omega}{c}|\mathbf{x}-\mathbf{x}'|}}{|\mathbf{x}-\mathbf{x}'|}\cdot\left(\frac{L}{v}\right)\mathbf{J}_{fi}(\mathbf{x}')\\
&= -\frac{i\hbar e}{2mvL^2\omega}\left\{\left(\frac{\omega}{c}\right)^2\overleftrightarrow{\mathbf{I}} + \nabla\nabla\right\}\cdot\left\{2\mathbf{k}_i - \mathbf{q}\right\}\int d\mathbf{x}'\,\frac{e^{i\frac{\omega}{c}|\mathbf{x}-\mathbf{x}'|}}{|\mathbf{x}-\mathbf{x}'|}e^{i\mathbf{q}\cdot\mathbf{x}'}\\
&= \frac{2i\pi\hbar e}{mvL^2\omega}\frac{e^{i\mathbf{q}\cdot\mathbf{x}}}{\left(\frac{\omega}{c}\right)^2 - q^2}\left\{\left(\frac{\omega}{c}\right)^2\overleftrightarrow{\mathbf{I}} - \mathbf{q}\mathbf{q}\right\}\cdot\left\{2\mathbf{k}_i - \mathbf{q}\right\}\\
&= \frac{2i\pi e\gamma_i}{k_iL^2\omega}\frac{e^{i\mathbf{q}\cdot\mathbf{x}}}{\left(\frac{\omega}{c}\right)^2 - q^2}\left\{\left(\frac{\omega}{c}\right)^2\overleftrightarrow{\mathbf{I}} - \mathbf{q}\mathbf{q}\right\}\cdot\left\{2\mathbf{k}_i - \mathbf{q}\right\}.
\end{aligned} \tag{26}$$

B. Localized Hermite-Gauss and Laguerre-Gauss Transverse States

We can now turn our attention to acquiring the effective transition current density and the resulting electric field it sources when the electron undergoes transitions between Laguerre-Gauss and Hermite-Gauss transverse states. In the weak-focusing limit, these modes take the form [41, 30, 48],

$$\begin{aligned}
\Psi^{\mathrm{HG}}_{n,m}(x,y) &= \frac{1}{w_0}\sqrt{\frac{2}{\pi n!m!}}\,2^{-\frac{n+m}{2}}H_n\left[\frac{x\sqrt{2}}{w_0}\right]H_m\left[\frac{y\sqrt{2}}{w_0}\right]e^{-\frac{x^2+y^2}{w_0^2}}\\
\Psi^{\mathrm{LG}}_{l,p}(R,\phi) &= \frac{1}{w_0}\sqrt{\frac{2p!}{\pi(|\ell|+p)!}}\left(\frac{\sqrt{2}R}{w_0}\right)^{|\ell|}\mathrm{L}^{(|\ell|)}_p\left[\frac{2R^2}{w_0^2}\right]e^{i\ell\phi}e^{-\frac{x^2+y^2}{w_0^2}}.
\end{aligned} \tag{27}$$

Due to the following recurrence relations involving the Hermite-Gauss functions, $H_{n+1}\left[\frac{x\sqrt{2}}{w_0}\right] = \frac{2x\sqrt{2}}{w_0}H_n\left[\frac{x\sqrt{2}}{w_0}\right] - \frac{w_0}{\sqrt{2}}H'_n\left[\frac{x\sqrt{2}}{w_0}\right]$ and $\frac{d}{dx}H_n\left[\frac{x\sqrt{2}}{w_0}\right] = \frac{2n\sqrt{2}}{w_0}H_{n-1}\left[\frac{x\sqrt{2}}{w_0}\right]$ one can derive a general expression for the derivative of an arbitrary Hermite-Gauss state $\partial_x\Psi^{\mathrm{HG}}_{n,m} = \frac{1}{w_0}\left(\sqrt{n}\Psi^{\mathrm{HG}}_{n-1,m} - \sqrt{n+1}\Psi^{\mathrm{HG}}_{n+1,m}\right)$ and $\partial_y\Psi^{\mathrm{HG}}_{n,m} = \frac{1}{w_0}\left(\sqrt{m}\Psi^{\mathrm{HG}}_{n,m-1} - \sqrt{m+1}\Psi^{\mathrm{HG}}_{n,m+1}\right)$. With this in mind, we can construct an effective transition current density for the transition between any two Hermite-

Gauss transverse electron states (units of statC·s^{-1} ·cm^{-2})

$$\mathbf{J}^{\mathrm{HG}}_{n,m\to n',m'}(\mathbf{x}) = -\frac{i\hbar e}{2mw_0L}\bigg(\big\{\sqrt{n}\Psi_{n-1,m}\Psi_{n',m'} - \sqrt{n+1}\Psi_{n+1,m}\Psi_{n',m'} - \sqrt{n'}\Psi_{n,m}\Psi_{n'-1,m'} + \sqrt{n'+1}\Psi_{n,m}\Psi_{n'+1,m'}\big\}\hat{\mathbf{x}}$$
$$+ \big\{\sqrt{m}\Psi_{n,m-1}\Psi_{n',m'} - \sqrt{m+1}\Psi_{n,m+1}\Psi_{n',m'} - \sqrt{m'}\Psi_{n,m}\Psi_{n',m'-1} + \sqrt{m'+1}\Psi_{n,m}\Psi_{n',m'+1}\big\}\hat{\mathbf{y}}$$
$$+ i\big\{k_i^z + k_f^z\big\}w_0\Psi_{n,m}\Psi_{n',m'}\hat{\mathbf{z}}\bigg)e^{iq_z z}. \tag{28}$$

Since the first order Hermite and Laguerre-Gauss states are related via $\Psi^{\mathrm{LG}}_{0,\pm1} = 1/\sqrt{2}\big\{\Psi^{\mathrm{HG}}_{10} \pm i\Psi^{\mathrm{HG}}_{01}\big\}$, we can construct a form for the effective transition current density involving first order Laguerre-Gauss states $\mathbf{J}^{\mathrm{LG}}_{0,\pm1\to00}(\mathbf{x})$ in terms of $\mathbf{J}^{\mathrm{HG}}_{10\to00}$ and $\mathbf{J}^{\mathrm{HG}}_{01\to00}$ as

$$\mathbf{J}^{\mathrm{LG}}_{0,\pm1\to00}(\mathbf{x}) = \frac{1}{\sqrt{2}}\Big\{\mathbf{J}^{\mathrm{HG}}_{10\to00}(\mathbf{x}) \pm i\mathbf{J}^{\mathrm{HG}}_{01\to00}(\mathbf{x})\Big\}. \tag{29}$$

Using these equations, we explicitly write out the transition current density for the various first order Hermite-Gauss and Laguerre-Gauss beams discussed in the main text

$$\begin{aligned}
\mathbf{J}^{\mathrm{HG}}_{00\to00}(\mathbf{x}) &= \frac{\hbar e}{2mL}\big|\Psi^{\mathrm{HG}}_{00}\big|^2(k_i + k_f)\hat{\mathbf{z}}e^{iq_z z}\\
\mathbf{J}^{\mathrm{HG}}_{10\to00}(\mathbf{x}) &= -\frac{i\hbar e}{mw_0L}\big|\Psi^{\mathrm{HG}}_{00}\big|^2\Big\{\hat{\mathbf{x}} + ix(k_i + k_f)\hat{\mathbf{z}}\Big\}e^{iq_z z}\\
\mathbf{J}^{\mathrm{HG}}_{01\to00}(\mathbf{x}) &= -\frac{i\hbar e}{mw_0L}\big|\Psi^{\mathrm{HG}}_{00}\big|^2\Big\{\hat{\mathbf{y}} + iy(k_i + k_f)\hat{\mathbf{z}}\Big\}e^{iq_z z}\\
\mathbf{J}^{\mathrm{LG}}_{0,\pm1\to00}(\mathbf{x}) &= -\frac{i\hbar e}{mw_0L}\big|\Psi^{\mathrm{HG}}_{00}\big|^2\,\Big\{\hat{\mathbf{x}} \pm i\hat{\mathbf{y}} + i(k_i + k_f)\big(x \pm iy\big)\hat{\mathbf{z}}\Big\}e^{iq_z z}.
\end{aligned} \tag{30}$$

To find analytic solutions for the fields of the OAM states, we need to know the forms for the Hermite-Gauss modes and the product of two Hermite-Gauss modes in the limit that $w_0 \to 0$. Following previous work [30, 48], and using the relation $\delta^{(n)}(x-x_0)\delta^{(n')}(x-x_0) = \frac{1}{w_0\sqrt{\pi}}\delta^{(n+n')}(x-x_0)$, one finds

$$\begin{aligned}
\lim_{w_0\to0^+}\Psi^{\mathrm{HG}}_{n,m} &= \frac{w_0\sqrt{\pi}}{n!m!}\left(\frac{-w_0}{2}\right)^{(n+m)}\delta^{(n)}(x-x_0)\delta^{(m)}(y-y_0)\\
\lim_{w_0\to0^+}(\Psi^{\mathrm{HG}}_{n',m'})^*\Psi^{\mathrm{HG}}_{n,m} &= \frac{1}{\sqrt{n!m!n'!m'!}}\left(\frac{-w_0}{2}\right)^{(n+m+n'+m')}\delta^{(n+n')}(x-x_0)\delta^{(m+m')}(y-y_0).
\end{aligned} \tag{31}$$

Using these limiting forms for the OAM state wavefunctions, the effective transition current densities take the form

$$\begin{aligned}
\mathbf{J}^{\mathrm{HG}}_{00\to00}(\mathbf{x}) &= \frac{\hbar e}{2mL}\delta^{(0)}(x-x_0)\delta^{(0)}(y-y_0)(k_i + k_f)\hat{\mathbf{z}}e^{iq_z z}\\
\mathbf{J}^{\mathrm{HG}}_{10\to00}(\mathbf{x}) &= -\frac{i\hbar e}{mw_0L}\Big\{\delta^{(0)}(x-x_0)\delta^{(0)}(y-y_0)\hat{\mathbf{x}} - \frac{iw_0^2}{4}\delta^{(1)}(x-x_0)\delta^{(0)}(y-y_0)(k_i + k_f)\hat{\mathbf{z}}\Big\}e^{iq_z z}\\
\mathbf{J}^{\mathrm{HG}}_{01\to00}(\mathbf{x}) &= -\frac{i\hbar e}{mw_0L}\Big\{\delta^{(0)}(x-x_0)\delta^{(0)}(y-y_0)\hat{\mathbf{y}} - \frac{iw_0^2}{4}\delta^{(0)}(x-x_0)\delta^{(1)}(y-y_0)(k_i + k_f)\hat{\mathbf{z}}\Big\}e^{iq_z z}\\
\mathbf{J}^{\mathrm{LG}}_{0,\pm1\to00}(\mathbf{x}) &= -\frac{i\hbar e}{mw_0L}\,\Big\{\delta^{(0)}(x-x_0)\delta^{(0)}(y-y_0)(\hat{\mathbf{x}} \pm i\hat{\mathbf{y}})\\
&\quad - \frac{iw_0^2}{4}(k_i + k_f)\big(\delta^{(1)}(x-x_0)\delta^{(0)}(y-y_0) \pm i\delta^{(0)}(x-x_0)\delta^{(1)}(y-y_0)\big)\hat{\mathbf{z}}\Big\}e^{iq_z z}.
\end{aligned} \tag{32}$$

Making use of the identity $\int dz' \frac{e^{i(\frac{\omega}{c})|\mathbf{x}-\mathbf{x}'|}}{|\mathbf{x}-\mathbf{x}'|} e^{\pm i q_z z'} = 2\mathrm{K}_0(\frac{q_z \Delta \mathbf{R}}{\gamma}) e^{\pm i q_z z}$, we can use the OAM transition current densities to find effective transition electromagnetic fields. For instance, the electric field for the $\Psi^{\mathrm{HG}}_{1,0} \to \Psi^{\mathrm{HG}}_{0,0}$ transition is

$$
\begin{aligned}
\mathbf{E}^0_{10\to00}(\mathbf{x},\omega) &= -4\pi i\omega \int d\mathbf{x}' \overleftrightarrow{\mathbf{G}}_0(\mathbf{x},\mathbf{x}',\omega)\cdot\Big(\frac{L}{v}\Big)\mathbf{J}^{\mathrm{HG}}_{10\to00}(\mathbf{x}') \\
&= \frac{i}{\omega}\int d\mathbf{x}' \Big\{\Big(\frac{\omega}{c}\Big)^2 \overleftrightarrow{\mathbf{I}} + \nabla\nabla\Big\} \frac{e^{i\frac{\omega}{c}|\mathbf{x}-\mathbf{x}'|}}{|\mathbf{x}-\mathbf{x}'|}\cdot\Big(\frac{L}{v}\Big)\mathbf{J}^{\mathrm{HG}}_{10\to00}(\mathbf{x}') \\
&= \frac{i}{\omega}\frac{-i\hbar e}{m w_0 v}\Big\{\Big(\frac{\omega}{c}\Big)^2 \overleftrightarrow{\mathbf{I}} + \nabla\nabla\Big\}\cdot\int d\mathbf{x}' \frac{e^{i\frac{\omega}{c}|\mathbf{x}-\mathbf{x}'|}e^{iq_z z'}}{|\mathbf{x}-\mathbf{x}'|}\Big\{\delta^{(0)}(x'-x_0)\delta^{(0)}(y'-y_0)\hat{\mathbf{x}} \\
&\qquad\qquad - \frac{iw_0^2}{4}\delta^{(1)}(x'-x_0)\delta^{(0)}(y'-y_0)(k_i+k_f)\hat{\mathbf{z}}\Big\} \\
&= \frac{ic}{\omega}\Big\{\Big(\frac{\omega}{c}\Big)^2 \overleftrightarrow{\mathbf{I}} + \nabla\nabla\Big\}\cdot\Big(\frac{-2i\hbar e}{m w_0 v c}\Big)\Big\{\mathrm{K}_0\Big(\frac{q_z\Delta\mathbf{R}_0}{\gamma}\Big)\hat{\mathbf{x}} + \frac{iqw_0^2}{4\gamma}(\Delta\hat{\mathbf{R}}_0\cdot\hat{\mathbf{x}})(k_i+k_f)\mathrm{K}_1\Big(\frac{q_z\Delta\mathbf{R}_0}{\gamma}\Big)\hat{\mathbf{z}}\Big\}e^{iq_z z} \\
&= \frac{ic}{\omega}\Big\{\Big(\frac{\omega}{c}\Big)^2 \overleftrightarrow{\mathbf{I}} + \nabla\nabla\Big\}\cdot\mathbf{A}^0_{10\to00}(\mathbf{x},\omega).
\end{aligned}
\tag{33}
$$

where we have defined an effective transition vector potential $\mathbf{A}^0_{10\to00}(\mathbf{x},\omega) = -\Big(\frac{2i\hbar e}{m w_0 v c}\Big)\Big\{\mathrm{K}_0\Big(\frac{q_z\Delta\mathbf{R}_0}{\gamma}\Big)\hat{\mathbf{x}} + \frac{iq_z w_0^2}{4\gamma}(\Delta\hat{\mathbf{R}}_0\cdot\hat{\mathbf{x}})(k_i+k_f)\mathrm{K}_1\Big(\frac{q_z\Delta\mathbf{R}_0}{\gamma}\Big)\hat{\mathbf{z}}\Big\}e^{iq_z z}$ in units of StatV·s and where the impact parameter is $\Delta\mathbf{R}_0 = |\mathbf{R}-\mathbf{R}_0|$. Repeating this process for the $\mathbf{J}^{\mathrm{HG}}_{00\to00}$ and $\mathbf{J}^{\mathrm{HG}}_{01\to00}$ electron state transitions, we find that (statV·cm^{-1}·s)

$$
\begin{aligned}
\mathbf{E}^0_{00\to00}(\mathbf{x},\omega) &= \frac{ic}{\omega}\Big\{\Big(\frac{\omega}{c}\Big)^2 \overleftrightarrow{\mathbf{I}} + \nabla\nabla\Big\}\cdot\Big(\frac{\hbar e}{mvc}\Big)(k_i+k_f)\mathrm{K}_0\Big(\frac{q_z\Delta\mathbf{R}_0}{\gamma}\Big)\hat{\mathbf{z}}\,e^{iq_z z} \\
\mathbf{E}^0_{10\to00}(\mathbf{x},\omega) &= \frac{ic}{\omega}\Big\{\Big(\frac{\omega}{c}\Big)^2 \overleftrightarrow{\mathbf{I}} + \nabla\nabla\Big\}\cdot\Big(\frac{-2i\hbar e}{m w_0 v c}\Big)\Big\{\mathrm{K}_0\Big(\frac{q_z\Delta\mathbf{R}_0}{\gamma}\Big)\hat{\mathbf{x}} + \frac{iq_z w_0^2}{4\gamma}(\Delta\hat{\mathbf{R}}_0\cdot\hat{\mathbf{x}})(k_i+k_f)\mathrm{K}_1\Big(\frac{q_z\Delta\mathbf{R}_0}{\gamma}\Big)\hat{\mathbf{z}}\Big\}e^{iq_z z} \\
\mathbf{E}^0_{01\to00}(\mathbf{x},\omega) &= \frac{ic}{\omega}\Big\{\Big(\frac{\omega}{c}\Big)^2 \overleftrightarrow{\mathbf{I}} + \nabla\nabla\Big\}\cdot\Big(\frac{-2i\hbar e}{m w_0 v c}\Big)\Big\{\mathrm{K}_0\Big(\frac{q_z\Delta\mathbf{R}_0}{\gamma}\Big)\hat{\mathbf{y}} + \frac{iq_z w_0^2}{4\gamma}(\Delta\hat{\mathbf{R}}_0\cdot\hat{\mathbf{y}})(k_i+k_f)\mathrm{K}_1\Big(\frac{q_z\Delta\mathbf{R}_0}{\gamma}\Big)\hat{\mathbf{z}}\Big\}e^{iq_z z}.
\end{aligned}
\tag{34}
$$

Due to the relation between the first order HG- and LG- states, (see Eq. (29) earlier in this section) linear combinations of $\mathbf{E}^0_{10\to00}(\mathbf{x},\omega)$ and $\mathbf{E}^0_{01\to00}(\mathbf{x},\omega)$ will produce the transition fields due to transitions between first order LG states $\mathbf{E}^0_{0,\pm1\to00}(\mathbf{x},\omega)$.

VI. RADIALLY AND AZIMUTHALLY POLARIZED $\mathbf{J}^{\perp}_{fi}(\mathbf{x})$

This section derives the transition current density $\mathbf{J}_{fi}(\mathbf{x})$ associated with an initial electron state $\Psi_i(\mathbf{x}) \propto J_{|\ell|}(k_\perp\rho)e^{i\ell\varphi}e^{ik_\parallel z}$ and a final electron state $\Psi_f(\mathbf{x}) \propto J_{|\ell'|}(k'_\perp\rho)e^{i\ell'\varphi}e^{ik'_\parallel z}$, where $J_{|\ell|}$ are first order Bessel functions with angular quantum number ℓ. The Bessel functions are solutions to the free particle Schrödinger equation in cylindrical coordinates and thus $\mathbf{J}_{fi}(\mathbf{x})$ will be expressed in cylindrical polar form. The gradient in cylindrical coordinates is $\nabla = \hat{\boldsymbol{\rho}}\frac{\partial}{\partial\rho} + \hat{\boldsymbol{\varphi}}\frac{1}{\rho}\frac{\partial}{\partial\varphi} + \hat{\mathbf{z}}\frac{\partial}{\partial z}$, where $\rho = \sqrt{x^2+y^2}$, with $x = \rho\cos\varphi$ and $y = \rho\sin\varphi$. In this coordinate system $\mathbf{J}_{fi}(\mathbf{x})$ takes the general form

$$
\begin{aligned}
\mathbf{J}_{fi}(\mathbf{x}) = \frac{i\hbar e}{2m}\Big\{&\Big[\Psi^*_f(\mathbf{x})\frac{\partial\Psi_i(\mathbf{x})}{\partial\rho} - \Psi_i(\mathbf{x})\frac{\partial\Psi^*_f(\mathbf{x})}{\partial\rho}\Big]\hat{\boldsymbol{\rho}} + \frac{1}{\rho}\Big[\Psi^*_f(\mathbf{x})\frac{\partial\Psi_i(\mathbf{x})}{\partial\varphi} - \Psi_i(\mathbf{x})\frac{\partial\Psi^*_f(\mathbf{x})}{\partial\varphi}\Big]\hat{\boldsymbol{\varphi}} \\
&+ \Big[\Psi^*_f(\mathbf{x})\frac{\partial\Psi_i(\mathbf{x})}{\partial z} - \Psi_i(\mathbf{x})\frac{\partial\Psi^*_f(\mathbf{x})}{\partial z}\Big]\hat{\mathbf{z}}\Big\}.
\end{aligned}
\tag{35}
$$

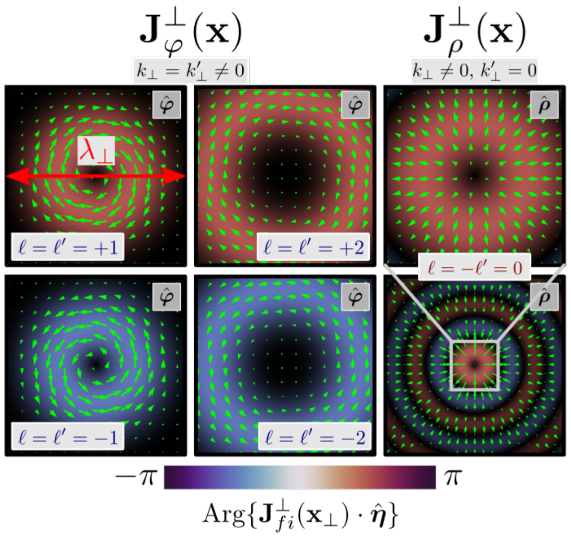

FIG. S4. **Pre- and post-selection of electron states in the cylindrical coordinate basis yielding perpendicular components of $\mathbf{J}_{fi}(\mathbf{x})$.** Left/center columns: The condition that $\ell = \ell'$ produces azimuthally polarized transition currents $\mathbf{J}^{\perp}_{\varphi}(\mathbf{x})$ with circular density profiles of increasing radius as $\ell = \ell'$ grows from ± 1 to ± 2 with $k_\perp = k'_\perp = 2\pi/\lambda_\perp$. Right column: Electron Bessel wave functions with $\ell' = -\ell = 0$, $k_\perp = 2\pi/\lambda_\perp$ and $k'_\perp = 0$ produce a radially polarized transition current $\mathbf{J}^{\perp}_{\rho}(\mathbf{x})$. Here the upper panel is a zoom in of the lower panel in the vicinity of the origin. In each case, the green arrows represent the direction of the vectorial flow of the current whereas the colors represent its complex phase projected onto the direction $\hat{\boldsymbol{\eta}} = \{\hat{\boldsymbol{\rho}}, \hat{\boldsymbol{\varphi}}\}$ as displayed in the upper right hand corner of each panel. The plotted width of each panel is $\lambda_\perp$ except for the lower right panel, which has a width of $4\lambda_\perp$.

Using the following recursion relations, $\frac{2\ell}{x} J_\ell(x) = J_{\ell-1}(x) + J_{\ell+1}(x)$ and $2\frac{d}{dx} J_\ell(x) = J_{\ell-1}(x) - J_{\ell+1}(x)$, and defining $\Delta\ell = \ell - \ell'$ and $q_\parallel = k_\parallel - k'_\parallel$, the transition current becomes

$$\begin{aligned}\mathbf{J}_{\ell,\,\ell',\,k_\perp,\,k'_\perp}(\mathbf{x}) = \frac{i\hbar e}{2m}\Big\{\Big[\frac{k_\perp}{2} J^*_{|\ell'|}(k'_\perp\rho)\big\{J_{|\ell|-1}(k_\perp\rho) - J_{|\ell|+1}(k_\perp\rho)\big\} - \frac{k'_\perp}{2} J_{|\ell|}(k_\perp\rho)\big\{J^*_{|\ell'|-1}(k'_\perp\rho) - J^*_{|\ell'|+1}(k'_\perp\rho)\big\}\Big]\hat{\boldsymbol{\rho}} \\ + \frac{i}{\rho} J^*_{|\ell'|}(k'_\perp\rho) J_{|\ell|}(k_\perp\rho)(\ell + \ell')\hat{\boldsymbol{\varphi}} + i J^*_{|\ell'|}(k'_\perp\rho) J_{|\ell|}(k_\perp\rho)(k_\parallel + k'_\parallel)\hat{\mathbf{z}}\Big\} e^{i\Delta\ell\varphi} e^{iq_\parallel z}.\end{aligned} \tag{36}$$

An azimuthally polarized transition current density can be constructed on the conditions that $\ell' = \ell$ and $k'_\perp = k_\perp$. In this case Eq. (36) reduces to the following,

$$\mathbf{J}_{\ell=\ell',\,k_\perp=k'_\perp}(\mathbf{x}) = -\frac{\hbar e}{2m}\big|J_{|\ell|}(k_\perp\rho)\big|^2\Big\{\frac{2\ell}{\rho}\hat{\boldsymbol{\varphi}} + (k_\parallel + k'_\parallel)\hat{\mathbf{z}}\Big\} e^{iq_\parallel z}, \tag{37}$$

which is a Bessel beam sourced by an electron moving moving in the $\hat{\mathbf{z}}$-direction. $\mathbf{J}_{\ell=\ell',\,k_\perp=k'_\perp}(\mathbf{x})$ is shown in the first and second columns of Fig. S4 for $\ell = \pm 1$ and $\ell = \pm 2$, respectively. Alternatively, if $\ell' = -\ell = 0$ and $k'_\perp = 0$ with $k_\perp \neq 0$, the current is radially polarized as

$$\begin{aligned}\mathbf{J}_{\ell'=-\ell'=0,\,k_\perp\neq 0,\,k'_\perp=0}(\mathbf{x}) &= \frac{i\hbar e}{4m}\Big\{k_\perp\big\{J_{-1}(k_\perp\rho) - J_1(k_\perp\rho)\big\}\hat{\boldsymbol{\rho}} + 2iJ_0(k_\perp\rho)(k_\parallel + k'_\parallel)\hat{\mathbf{z}}\Big\} e^{iq_\parallel z} \\ &= -\frac{i\hbar e}{2m}\Big\{k_\perp J_1(k_\perp\rho)\hat{\boldsymbol{\rho}} - iJ_0(k_\perp\rho)(k_\parallel + k'_\parallel)\hat{\mathbf{z}}\Big\} e^{iq_\parallel z},\end{aligned} \tag{38}$$

which is displayed in the third column of Fig. S4 at two different magnification levels.
